# The phase separation-dependent FUS interactome reveals nuclear and cytoplasmic function of liquid–liquid phase separation

**DOI:** 10.1093/nar/gkab582

**Published:** 2021-07-07

**Authors:** Stefan Reber, Daniel Jutzi, Helen Lindsay, Anny Devoy, Jonas Mechtersheimer, Brunno Rocha Levone, Michal Domanski, Eva Bentmann, Dorothee Dormann, Oliver Mühlemann, Silvia M L Barabino, Marc-David Ruepp

**Affiliations:** United Kingdom Dementia Research Institute Centre at King's College London, Institute of Psychiatry, Psychology and Neuroscience, King's College London, Maurice Wohl Clinical Neuroscience Institute, London, UK; United Kingdom Dementia Research Institute Centre at King's College London, Institute of Psychiatry, Psychology and Neuroscience, King's College London, Maurice Wohl Clinical Neuroscience Institute, London, UK; Department of Mathematics, École polytechnique fédérale de Lausanne (EPFL), Lausanne, Switzerland; United Kingdom Dementia Research Institute Centre at King's College London, Institute of Psychiatry, Psychology and Neuroscience, King's College London, Maurice Wohl Clinical Neuroscience Institute, London, UK; United Kingdom Dementia Research Institute Centre at King's College London, Institute of Psychiatry, Psychology and Neuroscience, King's College London, Maurice Wohl Clinical Neuroscience Institute, London, UK; Department of Biotechnology and Biosciences, University of Milano-Bicocca, Milan, Italy; Department of Chemistry and Biochemistry, University of Bern, Bern, Switzerland; Biomedical Center (BMC), Cell Biology, Ludwig Maximilians University Munich, Germany; Biomedical Center (BMC), Cell Biology, Ludwig Maximilians University Munich, Germany; Munich Cluster for Systems Neurology (SyNergy), Munich, Germany; Department of Chemistry and Biochemistry, University of Bern, Bern, Switzerland; Department of Biotechnology and Biosciences, University of Milano-Bicocca, Milan, Italy; United Kingdom Dementia Research Institute Centre at King's College London, Institute of Psychiatry, Psychology and Neuroscience, King's College London, Maurice Wohl Clinical Neuroscience Institute, London, UK

## Abstract

Liquid–liquid phase separation (LLPS) of proteins and RNAs has emerged as the driving force underlying the formation of membrane-less organelles. Such biomolecular condensates have various biological functions and have been linked to disease. The protein Fused in Sarcoma (FUS) undergoes LLPS and mutations in *FUS* have been causally linked to the motor neuron disease Amyotrophic Lateral Sclerosis (ALS-FUS). LLPS followed by aggregation of cytoplasmic FUS has been proposed to be a crucial disease mechanism. However, it is currently unclear how LLPS impacts the behaviour of FUS in cells, e.g. its interactome. Hence, we developed a method allowing for the purification of LLPS FUS-containing droplets from cell lysates. We observe substantial alterations in the interactome, depending on its biophysical state. While non-LLPS FUS interacts mainly with factors involved in pre-mRNA processing, LLPS FUS predominantly binds to proteins involved in chromatin remodelling and DNA damage repair. Interestingly, also mitochondrial factors are strongly enriched with LLPS FUS, providing a potential explanation for the observed changes in mitochondrial gene expression in mouse models of ALS-FUS. In summary, we present a methodology to investigate the interactomes of phase separating proteins and provide evidence that LLPS shapes the FUS interactome with implications for function and disease.

## INTRODUCTION

The biophysical process of liquid–liquid phase separation (LLPS) has drawn considerable attention over the last couple of years. Indeed, the regulators as well as the biophysical driving forces of LLPS are only just beginning to be understood. LLPS was not only reported to be important for the formation of various membraneless organelles such as nucleoli, P-bodies and stress granules, but also has been implicated in various diseases (1–3). One of the best studied proteins that undergoes LLPS *in vitro* and *in vivo* is Fused in Sarcoma (FUS) (4–10). FUS is a ubiquitously expressed RNA-binding protein that has been implicated in diverse RNA metabolic pathways, such as transcription, pre-mRNA splicing and miRNA processing (11–16). In 2009, mutations in *FUS* were shown to be causative for Amyotrophic Lateral Sclerosis (ALS) (17,18). ALS is the most common motor neuron disease in human adults and is characterized by a progressive loss of upper and lower motor neurons, causing paralysis and ultimately leading to death (19). The most frequent ALS-causing mutations in FUS disrupt its C-terminal nuclear localization signal (NLS), leading to cytoplasmic mislocalization and aggregation of FUS in neurons and glial cells of affected individuals, a pathological hallmark of FUS-ALS (20,21). Several recently published mouse models indicate a toxic gain of FUS function in the cytoplasm (22–24). Noteworthy, recruitment of FUS into phase separated RNP granules, e.g. stress granules, followed by aggregation has been proposed to drive disease (25,26).

Due to the current lack of tools to study membraneless organelles (27), LLPS-dependent FUS interactions are unknown and it is unclear how ALS-linked mutant FUS exerts its toxic function(s). Furthermore, it is also unknown if and how phase separation contributes to FUS function. Aiming to better understand the functional consequences of FUS phase separation and to identify FUS interactors under LLPS conditions, we developed a method that allows for the purification of phase separated FUS together with its associated proteins and RNAs, and compared these interactors to FUS interactors that were purified under non-LLPS conditions. We observed distinct interaction patterns depending on the biophysical state of FUS. Whereas FUS interacts predominantly with factors involved in RNA processing under non-LLPS conditions, phase separated FUS preferably interacts with proteins involved in chromatin remodeling, DNA damage response and proteins with functions in mitochondria. Furthermore, our data suggest that LLPS in different cellular compartments serves different functions. While phase separation-deficient FUS loses its association with chromatin and fails to autoregulate FUS expression, both LLPS-deficient and proficient ALS-linked mutant FUS affect cell viability to the same extent. Taken together, our findings indicate that LLPS is essential for some but not all physiological functions of FUS and LLPS may not be necessary for the gain-of-function toxicity of cytoplasmic mislocalized FUS. In summary, our method provides new insights into how LLPS affects FUS interaction partners and function. Notably, this method should be applicable to other proteins that undergo LLPS and allows to gain first insights into the role of LLPS in protein function and interactions.

## MATERIALS AND METHODS

### Oligonucleotides, plasmids and antibodies

Oligonucleotides, plasmids and antibodies are described in the Supplementary Information.

### Cell culture

HeLa and HEK293T cells were maintained in Dulbecco's modified Eagle's medium (DMEM) containing 10% fetal calf serum (FCS), penicillin (100 U/μl) and streptomycin (100 μg/ml)) at 37°C and 5% CO_2_. Cells were transfected using Dogtor (OZ Biosciences) for all experiments except for cytoplasmic/mitochondrial (membrane), soluble/insoluble fractionation experiments and transfections of HEK293T cells prior to stress followed by immunofluorescence. For these experiments, cells were transfected using TransIT-LT1 (Mirus). Moreover, for analysis of FUS stress granule localization after arsenite stress in HeLa cells, MTT assays and for the E1A minigene reporter assays, Lipofectamine 2000 (Thermo Fisher) was used according to the manufacturer's instructions.

### Generation of cell lysates for droplet purification and eGFP co-immunoprecipitation

30 μg plasmid coding for eGFP-GSG15-FUS or 15 μg plasmid coding for FLAG-eGFP, respectively were transiently transfected into 40–50% confluent HEK293T cells in a T300 flask using Dogtor according to the manufacturer's instructions. For the control experiment using the soluble GB1-TwinStrep-RFP as internal marker for global protein aggregation, 40 μg pcDNA6F-eGFP-FUS P525L and 10 μg pcDNA3.1-GB1-TwinStrep-RFP were transfected using Dogtor. The medium was replaced 24 h after transfection. 48 h after transfection, the cells were harvested by flushing with ice cold medium. Cells were pelleted at 4°C at 200 g for 5 min. After removal of the supernatant, the cells were washed with ice cold PBS. Thereafter, cells were pelleted at 4°C for 3 min at 1500 g. PBS was removed and the cells were snap frozen in liquid nitrogen and stored at –80°C until usage. The pellet was thawed on ice and re-suspended in the approximate volume of the cell pellet (400 μl) lysis buffer (75 mM HEPES pH7.3, 100 mM KOAc, 0.5 mM DTT, 0.5% NP40, 1:5000 Antifoam B, 10 μl/ml lysis buffer protease inhibitor, 10 μl/ml lysis buffer RNase inhibitor) and transferred to a 1.5 ml Eppendorf tube. To increase lysis efficiency, the cells were passed 5× through a 25 G 5/8 needle. The lysate was subsequently used to isolate FUS droplets or to perform an eGFP co-immunoprecipitation.

### Purification of FUS-containing droplets

To increase the formation of LLPS FUS droplets, the volume of the lysate was reduced to approximately half of the initial volume in a speedvac (to reach the approximate volume of the initial cell pellet). To reduce time in the speedvac, samples were split into several smaller samples of approx. 150 μl. To monitor droplet formation, a drop of the lysate was placed on a standard microscopy glass slide, covered with a coverslip and FUS droplets were visualized using a fluorescence microscope. Subsequently, 30 μl concentrated lysate were transferred to a new 1.5 ml Eppendorf tube and droplets were stabilized by adding 0.3 μl 10% formaldehyde (final conc. 0.1%). The lysate was mixed by vortexing and incubated for 8 min at room temperature. Remaining cross-linker was quenched through the addition of 1 μl 1 M TRIS pH7.3 and vortexing. The sample was thereafter stored on ice until droplets were sorted by fluorescence activated particle sorting (FAPS). Right before sorting, 300 μl PBS was added to the lysate and the sample was passed through a 40 μm cell strainer (to get rid of aggregates which could clog the sorter). Droplets were sorted into PBS according to eGFP-fluorescence and side scatter (SSC) on a FACS ARIA (BD Biosciences). Sorted droplets were stored at – 80°C until further processing. Before protein or RNA isolation, the droplets were pelleted by centrifugation at 4°C at 16 000 g for 15 min. The droplet-pellet was washed with 1 ml PBS. The wash was repeated one more time. Between washes, the droplets were centrifuged for 15 min 4°C at 16 000 g. After removal of the PBS, 150 000 droplets (sorted events) were either re-suspended in 50 μl 1× LDS-loading buffer (NuPAGE™ LDS Sample Buffer (4×) (NP0007, Thermo Fisher) diluted to 1× supplemented with 50 mM DTT) for protein elution or in 50 μl RNA-sample buffer (50 mM Tris pH7.0, 5 mM EDTA, 1% SDS, 10 mM DTT) for subsequent RNA isolation. Pictures and video of cell lysates and droplets were acquired using a wide-field fluorescence Leica DMI6000 B microscope.

### eGFP nanobodies and coupling to magnetic beads

Plasmids encoding for His-tagged anti-GFP nanobodies (clones LaG-9, LaG-16 and LaG-24) were obtained under the MTA from Michael P Rout laboratory (Rockefeller University, New York, USA). All three constructs were expressed in ArticExpress (DE3) cells (Agilent) and purified using Ni-NTA resin as described (28). Purified anti-GFP nanobodies were coupled to magnetic beads (Dynabeads M-270 Epoxy, Invitrogen) accordingly to manufacturer's instruction. The coupling mixture contained 20 μg of purified nanobody per 1 mg of beads. The coupling reaction was carried out (separately for each clone) with rotation, at 37°C for 20 h. Anti-GFP nanobodies coupled to magnetic beads were re-suspended in 50% glycerol/PBS (2 ml per 300 mg beads) and stored at –20°C. In order to increase GFP binding efficiency, beads coupled to three different clones were mixed together in 1:1:1 ratio.

### eGFP co-immunoprecipitation

20 μl GFP nanobodies coupled to magnetic beads were transferred to 1 ml lysis buffer (75 mM HEPES pH7.3, 100 mM KOAc, 0.5 mM DTT, 0.5% NP40, 1:5000 Antifoam B, 10 μl/ml lysis buffer protease inhibitor, 10 μl/ml lysis buffer RNase inhibitor). Lysis buffer was removed and washed again with 1 ml lysis buffer. After removal of the lysis buffer, the beads were re-suspended in 100 μl lysis buffer and added to 400 μl previously prepared cell lysate. The beads were incubated for 3 h at 4°C head over tail on a rotor. Thereafter, the supernatant was removed and the beads were washed 5 × 5 min in 1 ml of 2× lysis buffer at 4°C head over tail on a rotor. The first wash step contained protease and RNase inhibitors. After the last wash step, the beads were splitted in two parts and either re-suspended in 1 ml TRIZOL for RNA isolation or 50 μl 1× LDS-loading buffer for protein elution.

### Mass spectrometry

The formaldehyde crosslink was reversed, eGFP-fusion proteins were eluted from the beads by boiling the samples for 15 min at 95°C in LDS-loading buffer. Cell lysates mixed with equal volumes of 2× LDS-loading buffer and boiled for 15 min at 95°C served as input samples. To prepare the samples (each experimental condition in biological triplicates) for mass spectrometry, samples were run 1 cm into a 12% Bis-Tris Plus gel (Invitrogen). 150 000 droplets (sorted events) and 3/5 of the eGFP co-IP, respectively were loaded. The gel was subsequently washed 5 × 5 min in ultrapure water, 10 min in 0.1 M HCl and Coomassie stained for 2 h (0.12% (w/v) Coomassie G-250, 10% H_3_PO_4_ (v/v), 10% (w/v) NH_4_OAc, 20% MeOH (v/v)) to visualize proteins. The gel was de-stained for 8 × 30 min in ultrapure water. To prepare gel pieces for mass spectrometry, the gel was cut, using a clean scalpel, into approx. 0.25 cm horizontal bands. The bands were further cut into cubes of ∼1–3 mm^3^ and transferred to a 1.5 ml Eppendorf tube. Samples were stored at 4°C in 20% EtOH before reduction and alkylation. Samples were analyzed in a random order to avoid chromatographic batch effects. The gel pieces were reduced, alkylated and digested by trypsin as described elsewhere (29). The digests were analyzed by nano-liquid chromatography tandem masspectrometry (nLC-MS/MS) (EasyLC 1000 nanoflow-UPLC coupled to a QExactive HF mass spectrometer, ThermoFisher Scientific) with one injection of 5 μl digests. Peptides were trapped on a Precolumn (C18 PepMap100, 5 μm, 100 Å, 300 μm × 5 mm, Thermo Fisher Scientific, Reinach, Switzerland) and separated by backflush on a C18 column (3 μm, 100 Å, 75 μm × 15 cm, C18, Nikkyo Technos, Tokyo, Japan) by applying a 40-min gradient of 5% acetonitrile to 40% in water, 0.1% formic acid, at a flow rate of 350 nl/min. The Full Scan method was set with resolution at 60 000 with an automatic gain control (AGC) target of 1E06 and maximum ion injection time of 50 ms. The data-dependent method for precursor ion fragmentation was applied with the following settings: resolution 15 000, AGC of 1E05, maximum ion time of 110 ms, isolation width of 1.6 *m*/*z*, collision energy 27, under fill ratio 1%, charge exclusion of unassigned and 1+ ions, and peptide match preferred, respectively. Spectra interpretation was performed with MaxQuant/Andromeda version 1.5.4.1 searching against the forward and reversed SwissProt Homo Sapiens protein database (Release 2017_12) using fixed modification of carbamidomethylation on Cys, and variable modifications of oxidation on Met, deamidation on Asn /Gln, and acetylation on protein N-term. Mass error tolerance for parent ions was set to 10 ppm, fragment ion tolerance to 20 ppm, and full trypsin cleavage specificity with three missed cleavages were allowed. Based on reversed database matches a 1% false discovery rate (FDR) was set for acceptance of peptide spectrum matches (PSM), peptides, and proteins. Relative protein abundance was calculated as described elsewhere (30). In brief, contaminant proteins such as keratins and trypsin were removed and the remaining protein's iBAQ values were each divided by the sum of all non-contaminant iBAQ values. Relative iBAQ values were used to determine fold changes and *P*-values using Student's *t*-test and adjusted *P*-values (FDR) between experimental conditions. To be assigned to the LLPS interactome of FUS, proteins purified from FUS droplets had to be enriched >2-fold compared to the input with a FDR <0.05. To be assigned to the non-LLPS interactome, proteins co-IPed with FUS had to be enriched > 2-fold compared to the input as well as to the control IP (FLAG-eGFP), with a FDR <0.05 for both. The relative contribution of previously identified FUS interactors (12,31–36) to the FUS interactors identified in this study was calculates as follows: The sum of all FUS interactors that were identified in this study and in at least one of the above mentioned studies was divided by the sum of all FUS interactors identified in this study. The data is available in Supplementary Table S1.

### RNA isolation and RNA deep sequencing

To isolate RNA from the droplets, the formaldehyde crosslink was reversed through incubation of the droplets in RNA-sample buffer (50 mM Tris pH 7.0, 5 mM EDTA, 1% SDS, 10 mM DTT) for 40 min at 70°C. After cooling of the sample on ice for 5 min, 1 ml TRIZOL was added. RNA was isolated from TRIZOL according to the manufacturer's instructions. Quality and quantity of RNA was analyzed with an Agilent 2100 Bioanalyzer (Agilent Technologies). Total RNA isolated from transiently transfected cells served as input RNA. Total RNA isolated from droplets, from the co-IP and input RNA (in biological triplicates for each sample) were ribodepleted using the RiboMinus™ Transcriptome Isolation Kit (Invitrogen, K155004) before library preparation according to the manufacturer's manual. Libraries were prepared using the strand-specific Illumina TruSeq Stranded Total RNA kit (Part # 15031048 Rev. E). Total RNA libraries were sequenced on the Illumina HiSeQ3000 platform using 100 bp single-end sequencing cycles. Adapters and low quality bases were trimmed from reads using TrimGalore v0.4.4 https://github.com/FelixKrueger/TrimGalore (37,38). Reads were then mapped using Salmon v0.8.2 (39) to the human cDNA and non-coding RNA transcriptome, ENSEMBL version 38.90. Transcripts per million (TPM) were imported into R using tximport v1.4.0 (40), and differential gene expression analysis performed using edgeR v3.18.1 (41). Transcript lengths were included as an offset in modelling, and transcript length scaled TPMs were used for calculating average log_2_(TPM). RNAs with >2-fold change to the input with a FDR <0.001 were considered as significantly enriched over the input. To assess relative abundances of different RNA biotypes, several ENSEMBL gene biotypes were summarized in groups (pseudogenes include: *‘transcribed_unitary_pseudogene’, ‘unprocessed_pseudogene’, ‘processed_pseudogene’, ‘transcribed_unprocessed_pseudogene’, ‘polymorphic_pseudogene’, ‘transcribed_processed_pseudogene’, ‘IG_V_pseudogene’, ‘unitary_pseudogene’, ‘TR_V_pseudogene’, ‘TR_J_pseudogene’, ‘IG_C_pseudogene’, ‘IG_J_pseudogene’, ‘translated_processed_pseudogene’, ‘pseudogene’, ‘IG_pseudogene’*. lncRNAs include: *‘antisense_RNA’, ‘lincRNA’, ‘sense_intronic’, ‘sense_overlapping’, ‘bidirectional_promoter_lncRNA’, ‘3prime_overlapping_ncRNA’, ‘macro_lncRNA’*. other_ncRNA include: *‘processed_transcript’, ‘misc_RNA’, ‘ribozyme’, ‘sRNA’, ‘non_coding’*. other include: *‘TR_V_gene’, ‘IG_V_gene’, ‘IG_C_gene’, ‘IG_J_gene’, ‘TR_J_gene’, ‘TR_C_gene’, ‘IG_D_gene’ ‘TR_D_gene’*. 7SL and 7SK genes were excluded from the above groups to form each a separate group.

### STRING and gene ontology (GO) enrichment analysis

STRING (42) analysis was performed using the multi protein search function using default options. The ‘confidence’ option was chosen to indicate strength of data support for each interaction. In addition, GO terms indicated in the figure legends were highlighted. GO term analysis was performed using the WEB-based GEne SeT AnaLysis Toolkit (WebGestalt) (43) using default settings with the following options: organism of interest: *Homo sapien*s; method of interest: over-representation analysis (ORA); functional database: geneontology, biological process noRedundant. For GO term analysis of genes identified by mass spectrometry, the Reference Set ‘genome protein-coding’ was used. For GO term analysis of genes identified by RNAseq, the Reference Set ‘genome’ was used.

### Flow cytometry to analyze formation of FUS-droplets

To analyze FUS droplets by flow cytometry, FUS droplets were prepared as described above. Droplets were analyzed on a LSR II SORP H274 (BD Biosciences) and generated data was processed using FlowJo version 10.

### Soluble versus insoluble fractionations

60–80% confluent HEK293T cells in T25 flasks were harvested using Trypsin/EDTA and washed once with ice cold PBS. The cells were lysed in RIPA buffer (Thermo Fisher, 89900) containing 1× Halt™ Protease Inhibitor Cocktail (Thermo Fisher, 78429) for 20 min on ice with occasional vortexing. Samples were centrifuged for 15 min at 16 000 g at 4°C and the supernatant was subsequently mixed with equal volumes of 2× LDS-loading buffer (NuPAGE™ LDS Sample Buffer (4×) (NP0007, Thermo Fisher) diluted to 2× supplemented with 100 mM DTT) and boiled for 5 min at 95°C (soluble fraction). The insoluble pellet was washed twice with PBS and thereafter incubated in insoluble buffer (50 mM Tris pH7.5, 200 mM NaCl, 2 mM KCl, 1 mM EDTA, 0.5% Glycerol, 100 mM Urea, 1× Halt™ Protease Inhibitor Cocktail) for 1 h at 37°C at 1400 rpm. The sample was centrifuged for 5 min at 16 000 g and the supernatant was subsequently mixed with equal volumes of 2× LDS-loading buffer and boiled for 5 min at 95°C (insoluble fraction).

### Cytoplasmic/nucleoplasmic vs chromatin fractionation

40–60% confluent HEK293T cells on 10 cm plates were transfected with 3 μg expression construct (FLAG-FUS, FLAG-FUS PLD27YS or FLAG-FUS PLD27YS SV40NLS respectively) using Dogtor (OZ Biosciences) according to the manufacturer's instructions. The medium was changed 24 h after transfection and cells were harvested 48 h after transfection using a cell scraper. Cells were transferred to a 15 ml Falcon tube and spun for 5 min at 200 g at 4°C. After removal of the supernatant, cells were washed with 1 ml ice-cold PBS and transferred to a 1.5 ml Eppendorf tubed and pelleted for 3 min at 300 g at 4°C. Cells were re-suspended in 1 ml buffer A (10 mM HEPES pH7.9, 10 mM KCl, 1.5 mM MgCl_2_, 0.34 M sucrose, 10% glycerol, 0.1% Triton-X-100, 1 mM DTT and 1X Halt™ Protease Inhibitor Cocktail) and incubated for 5 min on ice. The suspension was centrifuged for 4 min at 1300 g at 4°C and the supernatant (cytoplasmic fraction) was transferred to a new Eppendorf tube. The pellet was washed once with buffer A. Thereafter, the pellet was re-suspended in 1 ml buffer B (3 mM EDTA, 0.2 mM EGTA, 1 mM DTT and 1× Halt™ Protease Inhibitor Cocktail) and incubated for 5 min on ice. The suspension was centrifuged for 4 min at 1700 g at 4°C and the supernatant (nucleoplasmic fraction) was combined with the cytoplasmic fraction (forming the cytoplasmic/nucleoplasmic fraction). The pellet was washed once with buffer B. Then, the pellet was re-suspended in 1 ml buffer C (50 mM Tris pH 7.5, 200 mM NaCl, 2 mM KCl, 1 mM EDTA, 0.5% glycerol, 100 mM urea and 1× Halt™ Protease Inhibitor Cocktail) and incubated in a heat block for 1 h at 37°C at 1400 rpm. After centrifugation for 5 min at 16 000 g, the supernatant (chromatin associated proteins) was transferred to a new Eppendorf tube. The pellet was subsequently washed with ultrapure water and buffer D (1 mM MgCl_2_, 1 mM CaCl_2_, 1× Halt™ Protease Inhibitor Cocktail). The pellet was then re-suspended in 884 μl buffer E (50 mM Tris pH 8.0, 10 mM NaCl, 1 mM MnSO_4_, 0.25 U/ml Cyanase (18542, Serva) and 1× Halt™ Protease Inhibitor Cocktail) and incubated for 15 min at 30°C at 600 rpm. Thereafter, NaCl was increased to 600 mM through addition of 116 μl of 5 M NaCl. The samples were incubated for another 15 min at 37°C at 1400 rpm. After centrifugation for 5 min at 16 000 g, the supernatant (chromatin) was combined with the chromatin associated fraction (forming chromatin fraction). Fractions were supplemented with equal amounts of 2× LDS-loading buffer and boiled for 5 min at 95°C.

### Cell viability assay – MTT

Two days prior to the experiment, HeLa WT were transiently transfected with either empty Flag vector, pcDNA6F-FUS P525L PLD27YS or pcDNA6F-FUS PLD27YS P525L constructs using Lipofectamine 2000 diluted in OptiMEM. After 18 h, cells were counted, and 8 × 10^3^ cells were seeded per well in 96-wells plates. Cells were allowed to attach for at least 24 h. The cells were then incubated for 4 h with a solution of MTT (3-(4,5-dimethylthiazol-2-yl)-2,5-diphenyltetrazolium bromide; Sigma-Aldrich) to a final concentration of 50 μg/ml. The solution was then removed, and cells were lysed in 100 μl DMSO before reading the absorbance (570 nm) using a multiwell VictorX spectrophotometer (Perkin-Elmer).

To assess the efficiency of transfected cells, some cells were fixed in 2% PFA and then immunostained for Flag. As the cells were in suspension, 3 min centrifugation at 300 g were done between each step. In brief, fixed cells were permeabilized in 0.1% Triton for 5 min and then washed twice in wash buffer (0.2% BSA in PBS). Cells were blocked in 20% FBS and 0.05% Tween20 diluted in PBS for 30 min, followed by two washes. Cells were incubated for 30 min with primary antibody mouse anti-Flag (1:150, Sigma-Aldrich F1804) diluted in wash buffer. After three washes, cells were incubated for 30 min with Alexa Fluor 488 goat anti-mouse (1:2000, Invitrogen A-11001). Cells were then washed twice with PBS and finally resuspended in 300 μl of PBS. Positive cells were quantified using a Beckman Cytoflex FACS apparatus. IBM SPSS version 26 software was used for statistical analysis. MTT data was analysed using one-way ANOVA. When appropriated, analysis was followed by Bonferroni post-hoc test for group-wise comparisons. Values of *P* < 0.05 were considered statistically significant.

### Immunofluorescence

HEK293T cells were grown on poly-d-lysine (Sigma Aldrich, A-003-E) coated eight-well slides (PEZGS0816, Milipore) for immunostaining experiments. For all experiments except for data presented in Figure 5E (see below), HeLa cells were grown in uncoated 8-well slides. Cells were fixed for 20 min in 4% PFA in PBS and subsequently washed 3× with PBS. For permeabilization and blocking, cells were incubated for 45 min in 0.5% Triton, 6% BSA in TBS at room temperature. Primary antibodies were diluted in 0.1% Triton, 6% BSA in TBS (TBS +/+) and added to the cells overnight at 4°C. Thereafter, cells were washed 3× with TBS +/+ and incubated for 1 h at room temperature with secondary antibodies diluted in TBS +/+. Cells were counterstained with DAPI (100 ng/ml in PBS) for 10 min at room temperature. After two additional wash steps in PBS, cells were mounted with Vecashield Hardset mounting medium (H-1400, Vector Laboratories). If indicated, the following stresses were applied prior to fixation. Arsenic stress: 0.5 mM sodium(meta)arsenite (NaAsO_2_) (S7400, Sigma Aldrich) for 1 h. Sorbitol stress: 0.4 M d-sorbitol (S1876, Sigma Aldrich). Slides were analyzed using a wide-field fluorescence Leica DMI6000 B microscope or a wide-field Ti-E epifluorescence microscope, Nikon.

For analysis of FUS stress granule localization after arsenite stress in Figure 5E, HeLa cells were seeded on coverslips in 24-well tissue culture plates and were transfected with 0.4 μg Flag-FUS-R495X or Flag-FUS R495X-PLD27YS using Lipofectamine 2000 according to the manufacturer's instructions. Twenty four hours later, cells were stressed with 0.5 mM Sodium (meta)arsenite (Sigma S71287) for 1 h and processed for immunocytochemistry as previously described (44), using anti-FLAG and anti-TIA-1 antibodies. Confocal images were obtained with an inverted laser scanning confocal microscope (Zeiss confocal 710) with a 63x/1.4 N.A. oil immersion lens. For quantification, the number of cells with TIA-1 positive SGs was counted in three independent experiments, analyzing at least 50 cells per experimental condition and replicate.

### Immunoblotting

Proteins from spinal cord of 9-month-old wild type and heterozygous Δ14 mice were generated as previously described (24). Protein lysates in LDS-loading buffer were separated on a NuPAGE 4–12% Bis–Tris Midi Gel (WG1403A or WG1402BOX, Thermo Fisher) and transferred on a nitrocellulose membrane using the iBlot Gel Transfer System (Thermo Fisher) according to the manufacturer's instructions. For analysis of SMARCA4 and SMARCA5, proteins were separated on a NuPAGE™ 3–8% Tris-Acetate Protein Gel (EA0375BOX, Thermo Fisher) and transferred on a nitrocellulose membrane using the iBlot™ 2 Gel Transfer Device (Thermo Fisher). Membranes were blocked in 2% BSA in in 0.1% Tween in Tris-buffered saline (TBST). Membranes were incubated with primary antibodies diluted in TBST for 2 h at room temperature. After 5 × 5 min wash steps in TBST, membranes were incubated with secondary antibodies in TBST for 1 h at room temperature. For immunodetection of SMARCA4 and SMARCA5, the SuperSignal™ Western Blot Enhancer kit (46640, Thermo Fisher) was used according to the manufacturer's instructions. To measure total protein levels from spinal cord lysates of mice, the REVERT™ Total Protein Stain Kit (926-11015, LI-COR) was used according to the manufacturer's instructions. The washed membranes were analyzed and signal intensity was determined (if required for the respective experiment) using the Odyssey Infrared Imaging System (LI-COR). Statistical significance of immunoblotting results was determined by paired t-test.

### FUS autoregulation, SCN4A minigene and E1A minigene reporter assay

To assess FUS autoregulation, 80% confluent HeLa cells in six wells were transfected with either 500 ng mock plasmid (control condition), pcDNA6F-FUS, pcDNA6F-FUS PLD27YS or pcDNA6F-FUS PLD27YS SV40NLS respectively. Twenty-four hours after transfection, cells were split into two wells of a 6-well plate. Seventy-two hours after transfection, cells were harvested (one-well into TRIZOL for RNA isolation, one well into 100 μl RIPA buffer containing 1× Halt™ Protease Inhibitor Cocktail. Before subsequent western blotting, the lysate was incubated for 20 min on ice, spun for 15 min at 4°C at 16 000 g and the supernatant was subsequently transferred to a new Eppendorf tube containing equal amount of 2X LDS-loading buffer and boiled for 5 min at 95°C.

For the SCN4A minigene reporter assay, 80% confluent HeLa cells in 6wells were transfected with 500 ng pSUPuro-scr or pSUPuro FUS for the CTR knockdown or the FUS knockdown, respectively. For the rescue condition, each 500 ng additional pcDNA6F-FUS, pcDNA6F-FUS PLD27YS or pcDNA6F-FUS PLD27YS SV40NLS were transfected. Twenty-four hoursx after transfection, the cells were split into two wells of a six-well plate and selection for the pSUPuro plasmids was started using 2 μg/ml Puromycin (CAS 58-58-2, Santa Cruz). Selection was maintained for 36 h. Cells were harvested 72 h after transfection as above (one well for RNA isolation, one well for western blotting).

For the E1A minigene reporter assay, 250 ng pcDNA6F-mEGFP, 500 ng pcDNA6F-FUS or 250 ng pcDNA6F-FUS PLD27YS SV40NLS along with 200 ng pEGFP-C1-E1A were transfected to HeLa cells in six-well plates at 80% confluency using Lipofectamine 2000 (11668019, Thermo Scientific). All plasmid mixes were filled up to 1 μg of total DNA using empty pcDNA3.1(+). Cells were harvested 48 hours post transfection and RNA isolation and western blotting was performed as described above.

### RT-qPCR

RNA was isolated from cells using TRIZOL (TRI Reagent™ Solution, AM9738, Thermo Fisher) supplemented with 1:100 β-Mercaptoethanol (A1108, PanReac Applichem) according to the manufacturer's instructions. RNA for the SCN4A reporter assay was DNase treated using the TURBO DNA-free™ Kit (AM1907, Thermo Fisher) prior to cDNA synthesis according to the manufacturer's instructions. RNA quantity was determined using the NanoDrop™ One/OneC Microvolume UV–Vis Spectrophotometer (Thermo Fisher). cDNA was generated from 1 μg RNA using the AffinityScript Multiple Temperature cDNA Synthesis Kit (00436, Agilent Technologies) according to the manufacturer's instructions using random hexamer primers (150 ng/μl) (Sigma Aldrich). To confirm successful DNase treatment, a control reaction omitting the reverse transcription enzyme was prepared. The cDNA was diluted to a RNA concentration of 8 ng/μl. qPCR was performed using the Takyon No ROX SYBR 2X MasterMix blue dTTP (UF-NSMT-B0701, Eurogentec) with a final MgCl_2_ concentration of 4 mM. For the E1A minigene reporter assay, 2 μg of RNA were reverse transcribed using the high-capacity RNA-to-cDNA kit (4387406, Applied Biosystems) according to the manufacturer's instructions. qPCR was performed using the MESA green no ROX SYBR 2× Master mix plus (RT-SY2X-06+NRWOU, Eurogentec). All samples were measured in technical duplicates: 3 μl of cDNA were amplified in a total volume of 15 μl containing each 600 nM forward and reverse primer using the Rotor-Gene Q 2plex Platform (Quiagen) with the following cycling parameters: 5 min 95°C (initial denaturation), 20 s 60°C, 5 s 95°C (40 cycles). After cycling, a melting curve was recorded from 65°C to 95°C rising by 1°C each step. Analysis was performed as described previously (45). Statistical significance of qPCR results was determined by unequal variances t-test using log-transformed splicing ratios.

### RNA FISH combined with immunofluorescence

HeLa cells were grown to 80% confluency in six-well plates and transfected with 1 μg of the FUS expression constructs. The next day, 40 000 transfected cells were re-seeded into eight-well slides (Merck, PEZGS0816) and incubated overnight. FISH/IF was essentially performed as described in (12). In brief, the cells were fixed with 4% PFA for 15 min, permeabilized in 70% Ethanol at 4°C for 48 h and blocked with blocking buffer (1% BSA (A7030, Sigma Aldrich) in PBS, supplemented with 2 mM Ribonucleoside Vanadyl Complexes (R3380, Sigma Aldrich). Antibodies were diluted in blocking buffer and incubated at room temperature for 1 hour (primary) and 2 h (secondary), respectively. Subsequently, antibody complexes were cross-linked to their targets using 4% PFA for 5 min. Following equilibration in 2× SSC (300 mM NaCl, 30 mM sodium citrate pH 7.0), and incubation in pre-hybridization buffer (15% Formamide (17899, Thermo Scientific), 10 mM sodium phosphate, 2 mM RVC in 2× SSC, pH 7.0) at 42°C for 10 min, 6-FAM azide labelled antisense probes were diluted to 0.5 ng/μl in hybridization buffer (15% Formamide, 10 mM sodium phosphate, 10% dextran sulfate (S4030, Merck), 0.2% BSA, 0.5 μg/μl *Escherichia coli* tRNA, 0.5 μg/μl salmon sperm DNA (15632011, Invitrogen), 2 mM RVC in 2× SSC, pH 7.0) and hybridized to their targets over night at 42°C. The next day, unbound probes were removed by washing two times 30 minutes with pre-hybridization buffer and three times 10 min in high stringency wash solution (20% Formamide, 2 mM RVC in 0.05× SSC, pH 7.0). Then, the cells were washed three times with 2× SSC before mounting with aqueous Vectashield mounting medium containing DAPI (H-1200, Vectorlabs).

### Silver staining

Proteins in LDS-loading buffer were separated on a NuPAGE 4–12% Bis–Tris Midi Gel (WG1403A, Thermo Fisher) or on a Bolt™ 4–12% Bis–Tris Plus Gel (NW04120BOX, Thermo Fisher). The gel was incubated for 2 h at room temperature in fixing solution (50% MeOH, 12% HAc, 0.05% formalin). The gel was subsequently washed 3 × 20 min at room temperature in 35% EtOH. Thereafter, the gel was sensitized in 0.02% Na_2_S_2_O_3_ for 2 min, washed 3 × 5 min in ultrapure water and incubated for 20 min at room temperature in silver staining solution (0.2% AgNO_3_, 0.076% formalin). Then, the gel was washed 2 × 1 min in ultrapure water and developed in developing solution (6% Na_2_CO_3_, 0.05% formalin, 0.0004% Na_2_S_2_O_3_). Upon desired band intensities, the reaction was stopped by replacing developing solution with stop solution (50% MeOH, 12% HAc) and incubating for 5 min.

## RESULTS

### Determining the liquid–liquid phase separation dependent FUS interactome

**Figure 1.**
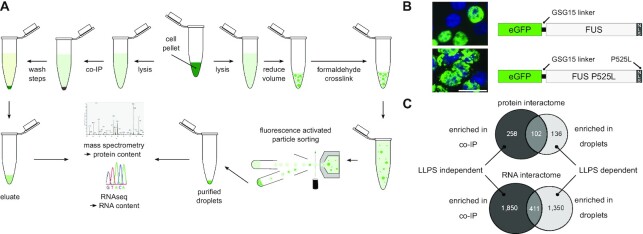
Co-IP and purification of LLPS FUS followed by quantitative mass spectrometry and RNA deep sequencing. (**A**) Experimental workflow. HEK293T cells expressing wild type or P525L eGFP-FUS fusion protein are lysed and subsequently subjected either to a co-IP experiment using anti-GFP nanobodies coupled to magnetic beads (left path) or to eGFP-FUS droplet purification (right path). Droplets are generated through reducing the volume of the lysate and stabilized using the reversible crosslinker formaldehyde. Thereafter, the droplets are purified by fluorescence-activated particle sorting and additional wash steps. (**B**) Constructs used for co-IP and droplet purification experiments. eGFP fused to FUS including a GSG15 linker between the two proteins. Wild type eGFP-FUS (top right) localizes mainly to the nucleus whereas ALS mutant P525L eGFP-FUS (bottom right) localizes predominantly to the cytoplasm as shown by fluorescence microscopy of transiently transfected HeLa cells counterstained with DAPI. Scale bar = 30 μm. (**C**) Summary of quantitative mass spectrometry (top) and RNA deep sequencing (bottom) experiments. Shown are numbers for protein and RNA species, which were significantly enriched in co-immunopurification and droplet purification experiments comprising the respective overlap between the two datasets.

In order to determine which protein and RNA species interact with FUS under LLPS conditions, we developed a novel approach which allows for the purification of phase separated FUS from cell lysates based on the purification of enhanced green fluorescent protein (eGFP)-tagged wild type FUS (summarized in Figure 1A). To identify possible alterations in the interactome of ALS-associated mutant FUS, we additionally employed eGFP-FUS P525L, an aggressive ALS-linked mutant where the C-terminal NLS is disrupted, leading to cytosolic mislocalization of FUS (21). To validate that both, eGFP-FUS and eGFP-FUS P525L, are capable of undergoing LLPS and forming liquid-like compartments in cells, we performed fluorescence recovery after photobleaching (FRAP) on HeLa FUS-KO cells transiently transfected with either FUS WT-GFP or FUS P525L-GFP and photobleached eGFP-FUS granules (nuclear for WT or cytoplasmic for P525L), and a nearby area to assess fluorescence recovery. We observed a fast recovery for both WT and P525L granules, reaching a maximum recovery within ∼20 s (Supplementary Figure S1a). Given that the hallmark of liquid-like structures is the dynamic reorganization and rapid exchange of proteins (46), our rapid recovery time is comparable to previous studies (4,47,48) and indicates that spontaneously generated FUS granules display liquid-like properties.

For our interactome studies, we transiently expressed FLAG-eGFP, eGFP-FUS and eGFP-FUS P525L in HEK293T cells (Figure 1B). It was previously shown that under physiological salt concentrations GFP-FUS starts to undergo LLPS *in vitro*, either through increasing concentrations above physiological concentrations of FUS or through the addition of a molecular crowding agent such as dextran (49). Following the same rationale to reach sufficient eGFP-FUS concentration for eGFP-FUS to undergo LLPS after cell lysis in the lysate, the volume of the cell lysate was concentrated 2-fold to the approximate volume of the initial cell pellet, thus restoring endogenous protein concentrations to near physiological levels. This volume reduction resulted in the formation of eGFP-FUS- and eGFP-FUS P525L-containing droplets in the cell lysate, which can be visualized by fluorescence microscopy (Supplementary Figure S1b, c, and Supplementary video). Importantly, no droplets were formed by FLAG-eGFP alone, confirming that FUS specifically drives phase separation (Supplementary Figure S1e). To further ensure that the concentration approach is not an inherent source of global protein aggregation, we co-expressed eGFP-FUS P525L with a soluble control protein (GB1-TwinStrep-RFP) and monitored droplet formation by fluorescence microscopy. While eGFP-FUS robustly formed droplets after volume reduction at an approximate concentration of 20 μM, GB1-TwinStrep-RFP remained dispersed and was not enriched in FUS-droplets even at 10-fold higher concentrations (Supplementary Figure S2a, b).

The eGFP-FUS droplets were then stabilized using the reversible crosslinker formaldehyde, which rendered them stable enough to be analysed by flow cytometry (Supplementary Figure S1e) and to be sorted by fluorescence activated particle sorting (FAPS) (50) (Supplementary Figure S1d). To address which protein and RNA species interact with FUS under non-LLPS conditions, a regular co-immunoprecipitation (co-IP) was performed using nanobodies against eGFP. As phase separation of FUS is highly dependent on FUS concentration (4,49) and the wash volumes applied during the co-IP exceeded the volume applied to analyse the cell lysates in Supplementary Figure S1e, LLPS of FUS during co-IP conditions is limited or even completely prevented. A pulldown of FLAG-eGFP served as control IP. Successful purification of the bait from droplet purifications and co-IPs were verified by SDS-PAGE followed by silver staining or western blotting, respectively (Supplementary Figure S3). Proteins and RNAs purified from co-IP and droplet purification experiments were analysed by label-free quantitative mass spectrometry or RNA deep sequencing, respectively. Interestingly, wild type and P525L FUS protein and RNA interactomes (under the same experimental conditions) were mostly identical (Supplementary Figure S4). This is most likely due to the fact that FUS and FUS P525L are both nuclear and cytoplasmic due to the ectopic overexpression (21,51). Therefore, wild type and P525L interactomes from the same experimental conditions were pooled, to increase statistical power for the identification of the most robust FUS interactors under LLPS and non-LLPS conditions. We identified 238 proteins interacting with FUS under LLPS conditions and 360 under non-LLPS conditions. 102 proteins were present in both datasets (independent of the biophysical state), resulting in 136 proteins specific for the LLPS condition. The observation that several proteins and RNAs preferentially interacted with FUS under LLPS conditions (Figure 1C and Supplementary Table S1) indicates that altered biophysical conditions within phase separated droplets enable FUS to undergo previously unknown interactions. Of note, while many proteins did not pass the statistical criteria (see material and methods) to be assigned to both interactomes, many proteins assigned to either the LLPS or the non-LLPS interactome were detected under both LLPS and non-LLPS conditions. Indeed, 70% of the LLPS interactors were detected in the non-LLPS condition, while 81% of the non-LLPS interactors were detected in the LLPS experiment. This indicates different binding, but not necessarily mutually exclusive binding to FUS, depending on its biophysical state. We further analysed proteins co-purified with FUS under LLPS and non-LLPS conditions, comparing them to previously reported FUS interactors (12,31–36). Interestingly, only 23.8% of the LLPS-dependent FUS interactors have been previously reported, whereas 47.2% of the co-immunoprecipitated FUS interactors have already been reported by these previous studies (12,31–36). This is in line with the idea that phase separation changes the biomolecular interactions of FUS. As aforementioned studies used non-LLPS conditions (co-immunoprecipitations) to purify FUS and its interaction partners, it is not surprising that there are clearly more previously unknown FUS interactors present in the LLPS-dependent FUS interactome.

While compared to the number of significantly enriched protein interactors, higher numbers of RNAs could be identified in FUS droplets (1761) or co-IPed together with FUS (2,261), with 411 RNAs significantly enriched in both samples (Figure 1C and Supplementary Table S2), the overall picture of the RNA interactome is similar to the protein interactome. More interactors were identified in the co-IP compared to the droplet purification experiment, and the relative overlap between the two samples is similar for both protein and RNA interactomes.

### FUS has a different protein interactome depending on its biophysical state

**Figure 2.**
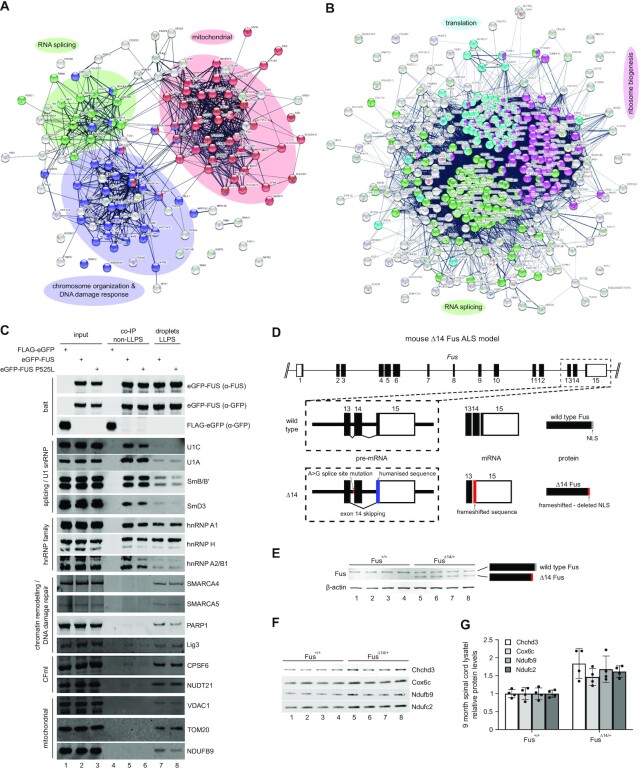
Preferential protein interaction partners depending on FUS biophysical state and dysregulated mitochondrial protein homeostasis at an early stage in FUS-ALS. (**A**) STRING analysis of LLPS-specific FUS interactors (*n* = 136). Highlighted are proteins with biological functions in RNA splicing (green, GO:0008380), chromosome organization and cellular response to DNA damage stimulus (blue, GO:0051276 and GO:0006974) and proteins mitochondrial functions (red, GO:0006839, GO:0007005 and GO:0006811). A high-resolution image is available in Supplementary Materials. (**B**) STRING analysis of non-LLPS interactors of FUS (*n* = 360). Highlighted are proteins with biological functions in RNA splicing (green, GO:0008380), translation (cyan, GO:0006412) and ribosome biogenesis (magenta, GO:0042254). A high-resolution image is available in Supplementary Materials. (**C**) Western blot analysis of proteins co-immunoprecipitated (non-LLPS) with control (FLAG-eGFP, lane 4) or FUS (lane 5–6) and purified together with FUS droplets (LLPS, lanes 7–8). (**D**) Scheme of the ‘FUSDelta14’ knockin mouse ALS model. A reported ALS mutation (FUS p.G466VfsX14) destroys the 3′ splice site of exon 14 leading to exon skipping resulting in a novel C-terminus deleting the endogenous FUS NLS. To generate the identical frameshift peptide to that of the human patient, human exon 15 coding sequence was also knocked-in. (**E**) Western blot analysis of spinal cord lysates from 9 month old FUS^+/+^ (lanes 1–4) and FUSΔ^14/+^ (lanes 5–8) mice. While FUS^+/+^ mice only express full-length Fus, FUSΔ^14/+^ mice express full-length and the shorter Δ14 Fus. β-actin served as loading control. (**F**) As in E, but showing changes in mitochondrial protein levels. Protein levels were normalized to total protein levels in each sample. (**G**) Quantification of western blot in F showing protein levels relative to wild type normalized to total protein levels.

In order to identify protein families enriched in LLPS and non-LLPS conditions, we performed a protein network analysis using STRING (42) on the 136 proteins that were exclusively co-purified with FUS under LLPS conditions and compared them to all the proteins (*n* = 360) that were co-immunoprecipitated together with FUS (non-LLPS conditions). Interestingly, STRING classified LLPS-specific FUS interactors into three main functional groups (Figure 2A), namely proteins with functions in mitochondria (red), proteins involved in chromatin remodelling and DNA damage response (blue) and proteins involved in RNA splicing (green). Although enriched in the LLPS-specific FUS interactome, proteins involved in RNA splicing were much more prominent among proteins co-immunoprecipitated together with FUS (Figure 2B, green). In line with this finding, performing gene ontology (GO) term enrichment analyses on the same groups of proteins using the WEB-based GEne SeT AnaLysis Toolkit (WebGestalt) (43) revealed that factors involved in RNA splicing and mRNA processing were enriched much more significantly in the non-LLPS FUS interactome compared to the LLPS-specific FUS interactome (Supplementary Figure S5a).

To validate the proteins detected by mass spectrometry, we performed western blot analysis of proteins involved in RNA splicing, chromatin remodelling and DNA damage repair and factors with functions in mitochondria (Figure 2C). While some proteins showed the same binding to FUS regardless of LLPS, such as hnRNP H and hnRNP A1, others showed a clear preference for either LLPS or non-LLPS conditions. Consistent with the mass spec data (Supplementary Table S1), proteins involved in mRNA splicing were co-purified with FUS independent of its biophysical state. Nonetheless, they seemed to interact with FUS preferentially under non-LLPS conditions. Furthermore, proteins involved in chromatin remodelling and DNA damage response as well as mitochondrial proteins were almost exclusively detectable together with phase separated FUS. Interestingly, nuclear FUS granules have already been reported to associate with RNA polymerase II (RNA Pol II), and the N-terminus of FUS, which can phase-separate and is also the transcriptional activator domain of FUS-CHOP and FUS-ERG fusion proteins observed in cancer, is sufficient to target the SWI/SNF chromatin remodelling complex (5,52–56). Moreover, chromatin remodelling is an important aspect of DNA damage response and FUS granules have already been reported at sites of DNA damage (4). In line with this data, proteins involved in the DNA damage response were specifically enriched with phase separated FUS. Strikingly, we also detected the members of mammalian cleavage factor I (CFIm) in our LLPS FUS interactome. Besides its function in mRNA 3′-end processing, CFIm has also been linked to chromatin remodelling (57) and is a component of paraspeckles which require FUS as structural component (58–61). The most unexpected and at the same time prominent LLPS-dependent FUS interactors, however, were proteins with function in mitochondria.

To ensure that the identified LLPS-dependent interactors are not artefacts from post-cell lysis protein rearrangements as well as formaldehyde crosslinking, we decided to further validate the interactions of FUS with TOM-20, VDAC1, SMARCA4 and CPSF6, which were only present in purified FUS droplets, as well as hnRNPH and hnRNPA2/B1, which were present both in the FUS co-IP and the FUS droplets. In our analysis we also included hnRNP K, which was significantly enriched in FUS droplets compared to the FUS co-IP (Supplementary Table S1) in order to analyse an hnRNP that displays selectivity to phase separated FUS. To preserve the phase separated state as well as to prevent post-lysis rearrangements, we performed *in situ* DSP/DTME crosslinking (62) of FLAG-tagged FUS P525L transfected 293T cells followed by immunoprecipitation from total extracts. Indeed, while hnRNP H and hnRNP A2/B1 co-precipitated in both, crosslinked or uncrosslinked conditions, hnRNP K as well as TOM-20 were only co-immunoprecipitated at a detectable level under crosslinked conditions, in line with their preference for phase separated FUS (Supplementary Figure S5a). CPSF6 and SMARCA4 however were insoluble in the IP buffer post-crosslinking (Supplementary Figure S5b), and VDAC1 which only showed a slight enrichment in the crosslinked IP compared to the uncrosslinked sample (Supplementary Figure S5a) showed a reduction in solubility post crosslinking (Supplementary Figure S5b). Hence, we performed proximity ligation assays for SMARCA4, CPSF6, VDAC1 as well as TOM-20 on HeLa cells transfected with either FLAG-FUS or FLAG-FUS P525L and confirmed their interaction with FUS in intact cells (Supplementary Figure S5c-e).

Consistent with previous reports showing that cytoplasmic FUS interacts with mitochondrial proteins and localizes into mitochondria (63–65), mitochondrial proteins formed the top GO term in the LLPS-specific FUS interactome (Supplementary Figure S6a). Intriguingly, it has previously been reported that dysregulation in mitochondrial gene expression occurs at the initial disease stage in ‘FUSDelta14’ knock-in mice, which heterozygously express FUS carrying a C-terminal frameshift mutation causing deletion of the NLS (24). To assess if mitochondrial protein levels are affected at an early, pre-symptomatic stage in the ‘FUSDelta14’ mouse model (Figure 2D and E), we quantified proteins isolated from spinal cord sections of pre-symptomatic mice by western blotting (Figure 2F and G). Normalization was performed using total protein staining (Supplementary Figure S6b) instead of relying on one single housekeeping gene. While proteins involved in RNA processing, DNA damage repair and chromatin remodelling appeared unchanged (Supplementary Figure S6c), several mitochondrial proteins were dysregulated in the ‘FUSDelta14’ knock-in mice. Of note, this was not due a general change of mitochondrial homeostasis, as other mitochondrial proteins remained unaffected (Supplementary Figure S6d). However, the exact mechanism for the observed dysregulation of a subset of mitochondrial proteins in the ‘FUSDelta14’ model and whether it is directly linked to increased interaction with LLPS FUS remains to be elucidated.

### FUS has a different RNA interactome depending on its biophysical state

**Figure 3.**
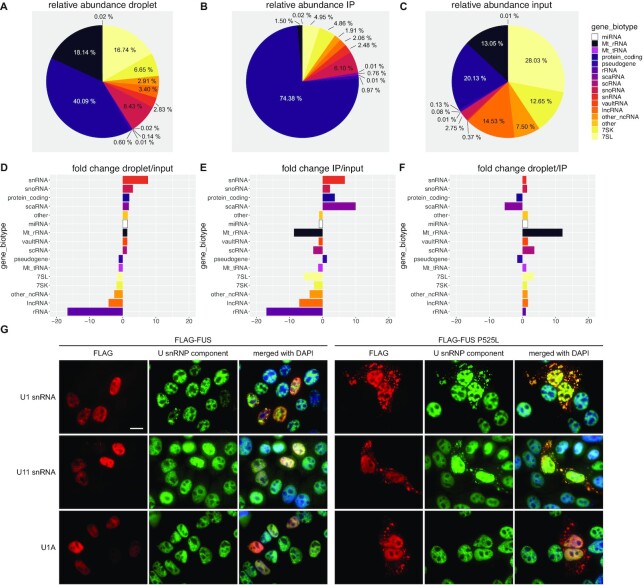
RNA interactome of LLPS and non-LLPS FUS. (**A**) Relative abundance of different RNA species co-purified with LLPS FUS (FUS droplets). (**B**) Relative abundance of different RNA species co-purified with non-LLPS FUS (co-IP). (**C**) Relative abundance of different RNA species in the input sample. (**D**) fold change of relative RNA abundance of different RNA species comparing RNAs found under LLPS conditions relative to the input. (**E**) Same as in D, but comparing RNA found in non-LLPS conditions compared to the input. (**F**) Same as in D, but comparing RNA abundance between LLPS and non-LLPS conditions. (**G**) HeLa cells transiently transfected with either FLAG-FUS (left) or FLAG-FUS P525L (right) and stained for FLAG (red channel) and different components of the U1 snRNP (green channel), either by RNA-FISH (U1 and U11, first and second row) or immunostaining (U1A, third row). Cells were counterstained using DAPI. Scale bar = 15 μm.

While mRNAs were the most abundant RNAs associated with either FUS droplets (LLPS conditions) or non-phase separated FUS (co-IP) (Figure 3A-C), the most prominently enriched RNA species co-purified with FUS under both conditions were U snRNAs (Figure 3D and E). U snRNAs are the RNA components of small nuclear ribonuclear particles (snRNPs) and are responsible for recognition and removal of introns from pre-mRNAs during splicing (66). Newly transcribed snRNAs are exported to the cytoplasm where a heptameric ring of Sm proteins is assembled onto them to generate a core snRNP. This core snRNP is then re-imported into the nucleus where snRNP-specific proteins (such as U1A in case of the U1 snRNP) assemble with the core snRNP to form the fully assembled, mature snRNP (67). Interestingly, in cells expressing FUS with NLS mutations the U1 core snRNP was found to mislocalize to the cytoplasm (68–70).

An enrichment for snRNAs was expected with non-phase separated FUS, as proteins involved in pre-mRNA splicing are also strongly enriched under non-LLPS conditions. However, the strong enrichment of snRNAs together with phase separated FUS was rather surprising, as snRNP protein components are not significantly enriched together with LLPS FUS. Interestingly, we and others have previously reported that cytoplasmic FUS granules sequester U snRNAs or SmB-associated U snRNAs, but not fully assembled snRNPs (12,68–70). Indeed, we could recapitulate that ALS-linked FUS P525L cytoplasmic foci bind U1 and U11 snRNAs in the cytoplasm, whereas the U1 snRNP specific protein U1A remains nuclear (Figure 3G). Together with our FRAP experiments (Supplementary Figure S1a) that indicate that cytoplasmic FUS P525L foci behave like phase separated bodies and our interactome data, this suggests that phase separated FUS in the cytoplasm preferentially binds partially assembled core snRNPs or unassembled snRNAs. Indeed, we and others have already shown that FUS contacts both the major (U2-type) and minor (U12-type) spliceosome to regulate splicing of specific introns (12,15,35). Finally, the biggest difference between the two purification experiments was the strong enrichment of mitochondrial ribosomal RNAs (MtrRNAs) (Figure 2F), which were strongly depleted in the non-LLPS condition, but clearly enriched in the LLPS condition. Importantly, this is consistent with the proteomic data, where we detected high levels of mitochondrial proteins purified together with FUS droplets, but not in the co-IP condition.

### LLPS is required for the association of FUS with chromatin and its function in autoregulation

**Figure 4.**
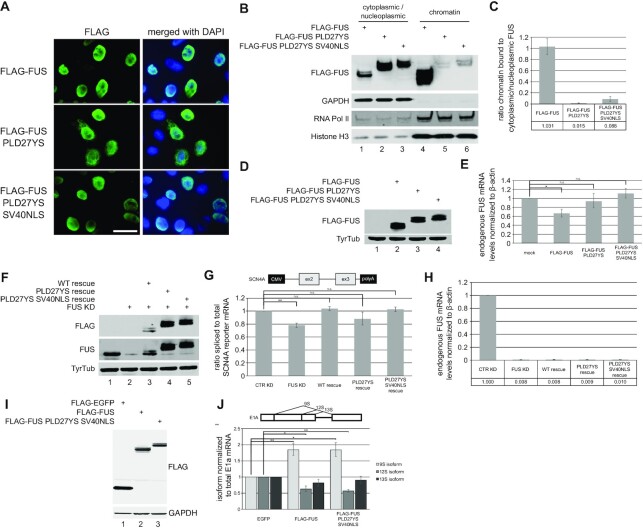
Phase separation is required for FUS binding to chromatin and function in FUS autoregulation. (**A**) Immunostaining of HeLa cells transiently expressing wild type FLAG-FUS, or LLPS-deficient FLAG-FUS PLD27YS and FLAG-FUS PLD27YS SV40NLS, respectively. Scale bar = 30 μm. (**B**) Western blot of cytoplasmic/nucleoplasmic versus chromatin fractionation experiment of HEK293T cells transiently expressing the constructs used in A. The blots were incubated with antibodies against FLAG. GAPDH, RNA Pol II and Histone H3 serve as controls for the respective fractions. While wild type FLAG-FUS is strongly bound to chromatin, phase separation deficient FUS is almost absent in the chromatin fraction. (**C**) Quantification of western blots in B. Shown is the ratio of chromatin bound to cytoplasmic/nucleoplasmic FUS relative to the wild type FLAG-FUS construct. Average values and standard deviation of three biological replicates are shown. (**D**) Western blot of HeLa cells which were either mock, FLAG-FUS, FLAG-FUS PLD27YS or FLAG-FUS PLD27YS SV40NLS transfected. Total cell lysates were subjected to SDS-PAGE and western blotting with antibodies against FLAG. Tyrosine tubulin served as loading control. (**E**) Endogenous FUS mRNA levels as determined by RT-qPCR relative to mock transfected cells. Average and standard deviations of three biological replicates are shown. Single asterisk indicates a *P*-values of <0.05. (**F**) Western blot analysis of FUS levels under control knockdown (CTR KD, lane 1), FUS KD (lane 2) and FUS rescue (lanes 3–5) conditions using wild type and LLPS-deficient FUS constructs. Proteins from HeLa extracts were separated by SDS-PAGE and blots were incubated with anti-FLAG (upper row) and anti-FUS (middle row). Tyrosine tubulin (lower row) serves as loading control. (**G**) Ratio of spliced to total RNA expressed from the SCN4A minigene (The minigene is driven by a CMV promoter and expresses exon 2 and 3 and the intervening U12-type intron) under CTR KD, FUS KD and FUS KD followed by a rescue with different RNAi-resistant expression constructs. Average values and standard deviations of three biological replicates are shown. Double asterisk indicates a *P*-values of <0.01. (**H**) Relative endogenous FUS mRNA levels from samples analysed in G. (**I**) Western blot of HeLa cells which were transfected with either FLAG-EGFP, FLAG-FUS, or FLAG-FUS PLD27YS SV40NLS. Total cell lysates were subjected to SDS-PAGE and western blotting with antibodies against FLAG. GAPDH served as loading control. (**J**) Ratios of 9S, 12S and 13S isoforms expressed from the E1A minigene under FLAG-EGFP, LLPS-proficient FLAG-FUS or LLPS-deficient FLAG-FUS PLD27YS SV40NLS overexpression. Average values and standard deviations of three biological replicates are shown. Single asterisk indicates a *P*-values of < 0.05, double asterisk indicates a *P*-value of <0.01.

In order to assess the importance of FUS LLPS for FUS function, we created an N-terminally FLAG-tagged FUS construct and substituted 27 tyrosines in the N-terminal prion-like domain (PLD) with serines (PLD27YS FUS). The aromatic ring structures of the tyrosines in the FUS PLD were previously shown to drive LLPS through inter-and intramolecular cation-π interactions with positively charged amino acid side chains (49,71). Mutating these tyrosines to serines abolishes phase separation *in vitro* and *in vivo* (5,49). Strikingly, PLD27YS FUS showed slightly increased cytoplasmic localization compared to wild type FUS (Figure 4A, first two rows). This suggests that besides the C-terminal NLS of FUS, also the N-terminus contributes to nuclear localization of FUS at steady state, possibly through phase-separation-dependent nuclear interactions of FUS. Indeed, it was previously reported that the N-terminus of FUS is required for binding of FUS to chromatin (72), where FUS localizes to granules and regulates gene expression in a transcription-dependent manner. Moreover, inhibition of transcription leads to cytoplasmic re-localization of FUS, suggesting that active transcription tethers FUS to newly synthesized RNA bound to chromatin (4,53,54). This is consistent with the recent finding that FUS leaves the nucleus through passive diffusion (73). If LLPS is indeed required for FUS binding to chromatin, one would expect more FUS diffusing from the nucleus to the cytoplasm if phase separation is inhibited. To be able to study the importance of LLPS for FUS function in the nucleus and to exclude that a loss of function is not due to FUS mislocalization, we generated a phase separation-deficient FUS construct with an additional strong SV40 NLS to ensure nuclear localization (Figure 4A, last row). To investigate the importance of LLPS for the ability of FUS to bind to chromatin, we transiently expressed wild type and LLPS-deficient FUS in HEK293T cells, followed by a cytoplasmic/nucleoplasmic versus chromatin biochemical fractionation. Indeed, LLPS-deficient FUS hardly interacted with chromatin compared to the wild type FUS (Figure 4B and C), indicating that phase separation is required for FUS function in co-transcriptional gene expression.

To test if LLPS-deficient FUS is still functional, we made use of three previously established assays for FUS function. First, we tested if PLD27YS FUS is still capable of autoregulating endogenous FUS mRNA levels when transiently transfected into HeLa cells (74). While wild type FUS autoregulated endogenous FUS mRNA levels, LLPS-deficient FUS had no effect on endogenous FUS mRNA levels (Figure 4D and E). Importantly, this was not due cytoplasmic mislocalization of PLD27YS FUS as the addition of the SV40 NLS did not rescue FUS function in autoregulation (Figure 4D and E). Next, we used the minor (also referred to as U12-type) intron containing SCN4A minigene, which requires FUS for efficient splicing (12). If cells are depleted of FUS, splicing of the SCN4A minigene becomes less efficient and this effect can be rescued by transient expression of RNAi-resistant FUS. Surprisingly, LLPS-deficient FUS was still able to promote efficient splicing (Figure 4F and G), even though it was unable to autoregulate. While PLD27YS FUS was only partially active, PLD27YS FUS harbouring the additional SV40 NLS fully rescued splicing of the SCN4A minigene. This difference presumably occurs due to the partial cytoplasmic mislocalization of PLD27YS FUS, which is rescued upon the addition of an additional NLS. Importantly, these differences did not emerge from differences in knockdown efficiencies of endogenous FUS, which are identical between the experimental conditions (Figure 4H). Finally, we used the well-established E1A minigene assay to assess the impact of LLPS in FUS’s function in major (U2-type) 5′ splice site selection. Overexpression of FUS was previously reported to increase the usage of a distal 5′ splice site on the E1A pre-mRNA resulting in an increased expression of the 9S isoform at the expense of proximal splice site selection, leading to reduced formation of the 12S and 13S isoforms (72,75–77). Indeed, we observed the same effect for wildtype FUS as well as LLPS-deficient FUS harbouring the additional SV40 NLS (Figure 4I and J), suggesting that LLPS is not required for FUS’s function in alternative 5′ splice site selection in the context of major intron splicing. The capability of LLPS-deficient FUS to promote splicing is in line with our observation that non-phase separated FUS preferentially interacts with proteins involved in RNA splicing, indicating that phase separation is not necessary for FUS function in splicing.

### Cytoplasmic FUS LLPS is not required for FUS toxicity, but for the formation of stress granules

**Figure 5.**
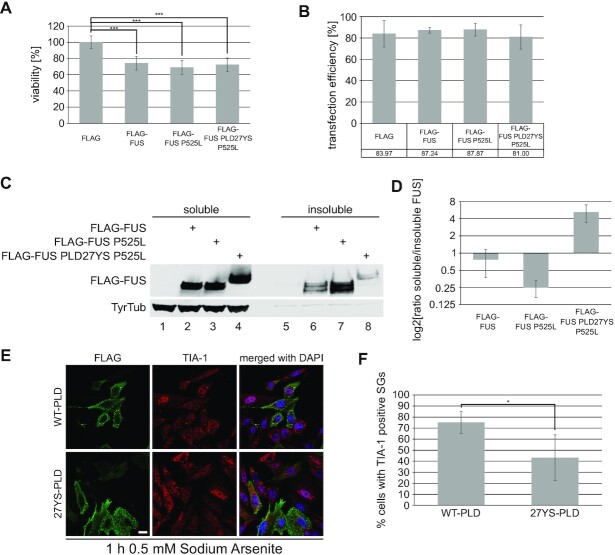
LLPS is not required for cytoplasmic FUS toxicity. (**A**) Effect of FLAG-FUS, FLAG-FUS P525L and FLAG-FUS PLD27YS P525L on cell viability compared to FLAG transfected control using the MTT assay. MTT data are expressed as the average viability in relation to FLAG control and were analysed using one-way ANOVA, followed by Bonferroni post-hoc test for group-wise comparisons. Two biological replicates with three technical replicates were performed (*n* = 6). Triple asterisks indicate a *P*-value <0.001. (**B**) Quantification of transfection efficiency of cells analysed in A. Transfected cells were immunostained against FLAG and positivity was assessed by FACS. (**C**) Western blot of soluble/insoluble fractionation of the cells transfected in A. Membranes were incubated with anti-FLAG antibodies. Tyrosine tubulin served as a loading control. (**D**) Quantification of FUS levels in (C). Shown is the ratio of soluble to insoluble FUS relative to wild type FLAG-FUS. Average values and standard deviation from five biological replicates are shown. (**E**) Immunostaining of arsenite-treated HeLa cells transiently transfected with LLPS-proficient (WT-PLD) or LLPS-deficient (27YS-PLD) FLAG-FUS R495X constructs lacking the C-terminal NLS. Cells were stained for Flag (green), the SG marker TIA-1 (red) and DAPI (blue). Scale bar = 20 μm. (**F**) Quantification of percentage of cells with TIA-1+ SGs (scored in at least 50 cells/experiment from three independent experiments), bar graph shows means and standard deviations. Average values and standard deviations from three biological replicates are shown. Asterisk indicates a *P*-value < 0.05 (Student's *t*-test, two sided).

It was previously reported that increased levels of FUS *in vivo*, either by overexpression or disruption of regulatory circuits, are associated with progressive motor neuron degeneration and ALS (51,78–80) and suggested that phase separation of FUS in the cytoplasm, followed by aggregation, drives disease (25,26). To assess whether LLPS of FUS is a prerequisite for the observed FUS toxicity, we performed MTT assays with HeLa cells transiently expressing FLAG peptide, FLAG-tagged wild type FUS, FUS P525L and LLPS-deficient FUS P525L (Figure 5A and B). As expected, overexpression of either wildtype or P525L FUS resulted in decreased cell viability in line with previous studies reporting that overexpression of FUS has deleterious effects in different models (51,78–80). Interestingly, LLPS-deficient FUS-P525L reduces cell viability to the same extent as LLPS-proficient FUS-P525L. To corroborate these results, we also assessed release of cytochrome c from mitochondria to the cytoplasm, a key step during the mitochondrial apoptotic pathway (81) (Supplementary Figure S7a, b). To assess the impact of LLPS on this pathway we transfected 293T cells either with empty pcDNA3.1(+) as control, FUS P525L and LLPS-deficient FUS P525L. Overexpression of either wild type or mutant FUS caused an increase in cytochrome c release, and in line with the results from the cell viability assay, LLPS-deficient FUS did not result in a reduction of released cytochrome c (Supplementary Figure S7a-b). To exclude potential toxic effects of the transfection reagent used for this experiment, untreated cells were compared to mock transfected cells, excluding a toxic effect of the transfection reagent (Supplementary Figure S6c and d). Overall, these data indicate that LLPS is not a prerequisite for FUS to exert deleterious effects on cells upon overexpression.

As FUS aggregates were suggested to exert a toxic function, we compared the solubility of LLPS-competent and LLPS-deficient FUS. To this end, half of the cells transfected for the mitochondrial/cytoplasmic fractionation were subjected to a RIPA soluble/insoluble biochemical fractionation experiment. As previously reported (82), ALS mutant cytoplasmic FUS exhibited reduced solubility compared to wild type FUS (Figure 5C and D). Interestingly, LLPS-deficient cytoplasmic FUS was more soluble than both wild type and cytoplasmic LLPS-competent FUS. Indeed, it was already shown, using purified FUS protein *in vitro*, that phase separation precedes the formation of insoluble aggregates (4). Our data strongly suggests that also in more complex environments, such as cell systems and *in vivo*, phase separation is involved in the formation of insoluble FUS aggregates, and therefore may be the mechanism by which FUS aggregates observed in post-mortem tissue of ALS-FUS patients are formed.

It has been postulated that recruitment of FUS to stress granules (SGs) may be a first step in the formation of insoluble FUS aggregates (20,21). Indeed, SGs are phase separated cytoplasmic compartments (83), and various studies have reported FUS localization to SGs, in particular when FUS is mislocalized to the cytoplasm due to a NLS mutation (21,84–86). In order to investigate the effect of LLPS on SG recruitment of FUS, we transiently transfected HeLa cells with NLS-deficient FUS R495X or FUS R495X PLD27YS (LLPS-deficient), induced SGs using sodium arsenite and then monitored SG recruitment of FUS using fluorescence microscopy. As expected, NLS-deficient cytosolically mislocalized FUS was efficiently recruited to TIA-1-positive SGs (Figure 5E), as previously reported (21,44,86). In contrast, LLPS-deficient cytosolic FUS remained mainly diffuse and very few TIA-1-positive SGs were seen in FUS-PLD27YS-expressing cells upon exposure to sodium arsenite stress (Figure 5E, see F for quantification), suggesting that SG formation is suppressed by LLPS-deficient FUS. This is a surprising finding, given that SG formation does not require FUS (87). To exclude the possibility that LLPS-deficient FUS only prevents TIA-1 from being recruited to SGs, but not SG formation *per se*, we performed the same experiment with immunostaining for other well-defined SG markers in HEK293T cells (TIAR and G3BP1). As observed for TIA-1, these other SG marker proteins also showed reduced localization to granular structures after arsenite stress in cells expressing LLPS-deficient cytosolic FUS (Supplementary Figure S8a and b), suggesting that SG formation may indeed be reduced by the presence of LLPS-deficient FUS. To further exclude that this behaviour is specific to arsenite-induced oxidative stress, we also applied osmotic stress to HEK293T cells transiently expressing the aforementioned FUS constructs using d-sorbitol. Consistent with the oxidative stress condition, LLPS-deficient FUS also reduced the formation of SGs under osmotic stress (Supplementary Figure S8c). Two possible explanations for this behaviour are: Either LLPS-deficient FUS protects cells from oxidative and osmotic stress, or LLPS-deficient FUS prevents SG formation through sequestration of factors required for this process. Although the first possibility cannot be excluded at this point, it seems rather unlikely, especially since LLPS-deficient FUS affects cell viability to the same extent as LLPS-competent FUS in our experiments. Interestingly, we (Figure 2 and Supplementary Table S1) and others (33–35,86) identified interactions between FUS and different SG marker proteins. Hence, we suggest that LLPS-deficient FUS might sequester factors required for SG formation and thus may prevent their incorporation into SGs during stress, thereby affecting SG assembly.

In summary, we provide evidence that LLPS and aggregation of FUS are not required to exert cellular toxicity upon FUS overexpression. Nonetheless, LLPS of FUS seems to be important for the formation of SGs and recruitment of FUS to these membrane-less organelles, a process that may proceed to the formation of insoluble FUS aggregates.

## DISCUSSION

In this study, we describe a novel method that allows for the purification of liquid–liquid phase separated proteins combining chemical crosslinking with fluorescence-activated particle sorting. Like every experimental approach, also this method has limitations which should be considered. Similar to co-immunoprecipitation experiments where post lysis rearrangements have been described (88), re-arrangements or interactions that are not occurring under physiological conditions might also arise following this approach when purifying liquid–liquid phase separated proteins. Hence, performing orthogonal assays such as *in situ* DSP/DTME crosslink followed by immunoprecipitation, proximity ligation assays and functional experimental readouts are essential in order to validate newly identified interactions by our LLPS droplet purification method. Moreover, including a fluorescent control can help to detect global aggregation which should be avoided as it would trap proteins and RNAs in a non-specific manner. Finally, normalization of the co-purified proteins and RNAs to their respective input abundance is crucial for the interpretation of the LLPS dependent interactome data. As for CLIP-Seq experiments where read counts depend on the expression level of a transcript making normalization to input RNA levels a critical step in the CLIP-Seq workflow (89), highly abundant protein and RNA species are expected to be crosslinked to the bait protein following the LLPS droplet purification workflow by chance. Interactors that bind preferably or exclusively under phase separating conditions are expected to be significantly enriched in the LLPS interactome compared to the input.

This approach allowed us to identify new and validate previously reported FUS interactors. In order to validate previously unknown interactions, we performed orthogonal assays to confirm that these interactions occur under physiological conditions. We show that LLPS changes the FUS interactome, presumably due to altered local concentrations of FUS and its interactors, favouring these interactions in phase separated compartments. Nevertheless, many FUS interactors are detected under both conditions but show a clear preference for either dispersed/soluble or phase separated FUS. Importantly, several proteins enriched with phase separated FUS, such as DDX3X, DHX9, FMR1, TIA-1 and SMN1 (compare Supplementary Table S1), have already been observed by others to co-localize with FUS into cytoplasmic granules (33,34,86,90). Moreover, proteins found in nuclear granules, specifically paraspeckles (61) and transcription-dependent granules containing FUS and RNA Pol II (53), are highly enriched under LLPS conditions compared to non-LLPS conditions (compare data in Supplementary Table S1).

We identified factors involved in chromatin remodelling and DNA damage repair to be the most prominent nuclear protein families binding with high preference to LLPS FUS. Interestingly, it has been observed that LLPS occurs at site of DNA damage (91,92). Strikingly, FUS was recently shown to be required for the correct recruitment of DNA damage repair factors to sites of DNA damage. Importantly, this process is dependent on FUS-induced LLPS, which is required for the recruitment of SFPQ, as LLPS-deficient FUS failed to recruit SFPQ to sites of DNA damage (93). Of note, SFPQ is one of most enriched proteins identified in our LLPS-dependent FUS interactome (Supplementary Table S1). Besides LLPS emerging as an important factor in DNA damage response, numerous recent studies reported that transcription factors recruit the mediator coactivator complex through phase separation using their activation domains leading to recruitment of RNA Pol II binding RNA Pol II C-terminal domain (CTD) (48,94–97). Noteworthy, FUS was previously reported to interact with the CTD of RNA Pol II (13) and was identified in transcription-dependent granules together with RNA Pol II (53). Moreover, the N-terminal domain of FUS, which was identified as the transcriptional activator in FUS-CHOP and FUS-ERG fusion proteins in liposarcoma and myeloid sarcoma respectively (54,55), was recently shown to be sufficient to contact the SWI/SNF chromatin remodelling complex (52). Strikingly, protein components of the mediator complex as well as protein subunits of RNAP II detected by mass spectrometry are clearly more abundant under LLPS conditions. Indeed, many of these proteins were exclusively detected in the LLPS condition while they were completely absent under non-LLPS conditions (Supplementary Figure S9). In addition, components of the mammalian pre-mRNA 3′ end processing factor CFIm (composed of NUDT21 (also CPSF5), CPSF6 and CPSF7) which were linked to chromatin remodelling (57) and paraspeckles (58), are more strongly enriched together with LLPS FUS compared to non-LLPS FUS, while in contrast, the other members of the 3′-end processing machinery have slightly higher enrichment under non-LLPS conditions (Supplementary Figure S10). In line with the idea that phase separation of FUS plays an important role in its chromatin-associated function(s), we show that FUS requires LLPS to efficiently bind to chromatin. Consistently, LLPS-deficient FUS mostly dissociates from chromatin and partially re-localizes to the cytoplasm. Most likely, LLPS FUS binds to chromatin in a transcription-dependent manner, since previous studies reported re-localization of FUS to the cytoplasm upon transcription inhibition (4,54), and FUS was found in transcription-dependent granules together with RNA Pol II (53). Our data further suggests that LLPS is the driving force for FUS binding to chromatin. The cytoplasmic re-localization of LLPS-deficient FUS, as a consequence of losing its nuclear tether, is consistent with recent data showing that the prion-like domain of FUS is required for chromatin association (72), and that FUS predominantly exits the nucleus through passive diffusion (73). In addition, we show that LLPS-deficient FUS can no longer exert its autoregulatory function, suggesting that during transcription FUS is recruited through LLPS to the *FUS* gene to regulate its own expression.

In contrast, non-LLPS conditions facilitate the interaction between FUS and splicing factors. Moreover, LLPS is not required to promote splicing of the minor intron containing SCN4A reporter gene or for alternative 5′ splice site selection in the E1A reporter gene. Of note, it has recently been shown that TDP-43, another protein of the hnRNP family that undergoes LLPS, does not to require phase separation to perform its function in pre-mRNA splicing (98). This is in line with studies which performed *in vitro* splicing assays in dependence on FUS or TDP-43, respectively (14,99). Both studies used HeLa nuclear extracts where nuclear components are highly diluted (compared to the *in vivo* context) and thus phase separation of either FUS or TDP-43 very unlikely. Nonetheless, both proteins function in splicing in the *in vitro* context. Altogether, these findings strongly indicate that LLPS is not required for the function of neither FUS nor TDP-43 in pre-mRNA splicing.

Besides providing evidence for the importance of LLPS for FUS nuclear function in autoregulation, our data suggests that LLPS and aggregation of FUS are not a prerequisite for reduced cell viability or increased apoptosis, at least upon overexpression in *in vitro* experiments. Of note, recent ALS-FUS mouse models, including the FUSDelta14 mouse that express cytoplasmic FUS from the endogenous mouse locus or at endogenous levels, consistently showed motor neuron degeneration in the absence of FUS aggregation. Indeed, all of these studies failed to detect cytoplasmic FUS inclusion bodies which are observed in human tissue (22–24). Although toxicity of cytoplasmic FUS aggregates cannot be excluded at this point, these findings strongly indicate that increased cytoplasmic concentrations of FUS are sufficient for having deleterious effects, leading to motor neuron death. Moreover, our data suggests that FUS LLPS is not necessary for the toxic effects of cytoplasmic FUS. It is therefore tempting to speculate that under physiological conditions the formation of insoluble cytoplasmic FUS inclusions could be a mechanism how cells reduce the amounts of cytoplasmic FUS, namely through locally concentrating FUS, leading to FUS LLPS and subsequent recruitment to SGs, followed by precipitation through a liquid-to-solid state transition. Thereby, FUS-LLPS might be a cellular mechanism to prevent (or reduce) deleterious effects, reducing the amount of soluble (toxic) FUS in the cytoplasm. Of note, this is not a new concept in the field of neurodegeneration: similarly, amyloid plaques in Alzheimer's disease have been proposed to buffer toxic soluble amyloid-beta species (100). While it remains to be established if LLPS and aggregation are dispensable for cytoplasmic FUS toxicity *in vivo*, our data provides additional evidence that LLPS may indeed be the precursor of insoluble FUS aggregates, as LLPS-deficient FUS is clearly more soluble than LLPS-competent FUS when overexpressed in HEK293T cells. This indicates that, similar to *in vitro* studies (4), also in cells LLPS is a prerequisite for liquid-to-solid state transition of FUS leading to the formation of insoluble FUS aggregates. Additionally, while LLPS-competent FUS robustly localizes to stress granules, LLPS-deficient FUS does not localize to oxidative or osmotic stress-induced stress granules and appears to even suppress stress granule formation. This may be due to aberrant interactions of LLPS-deficient FUS with key SG proteins, thereby interfering with SG nucleation. Alternatively, LLPS-deficient FUS may destabilize certain interactions within SGs and thus cause enhanced SG dissolution. In any case, our data suggest that the formation of SGs is not necessary for toxicity of cytoplasmic FUS, nor does SG formation protect against detrimental effects seen upon transient FUS overexpression.

To conclude, we find that LLPS alters and expands the interactome and functions of FUS. Furthermore, our data suggest that LLPS in the cytoplasm is not a prerequisite for FUS to exert detrimental effects on cells. The novel approach that we describe, which allows the identification of LLPS-specific protein and RNA interactors, should be applicable to other proteins undergoing LLPS and therefore will be a useful method for investigating how LLPS affects protein/RNA interactions and functions of phase-separating proteins.

## DATA AVAILABILITY

The mass spectrometry data are available via ProteomeXchange with the identifier PXD015834.

The high-throughput sequencing data are available via Array Express with the accession number E-MTAB-8456.

Flow Cytometry data are available via FlowRepository with the identifier FR-FCM-Z35F.

## Supplementary Material

gkab582_Supplemental_Files

## Notes

Present address: Stefan Reber, Skyhawk Therapeutics Europe GmbH, Basel, Hochbergerstrasse 60C, CH-4057, Switzerland.

Present address: Dorothee Dormann, Johannes Gutenberg University and Institute of Molecular Biology, Mainz, Germany.
