# Supplementary material for: The phase separation-dependent FUS interactome reveals nuclear and cytoplasmic function of liquid–liquid phase separation
Source: Nucleic Acids Res. 2021 Jul 7;49(13):7713–31. doi: 10.1093/nar/gkab582 (PMC8287939; doi:10.1093/nar/gkab582)
Supplement: gkab582_Supplemental_Files [file gkab582_supplemental_files.zip › Supplementary Materials revised.pdf]

Supplemental Material, Methods and Figures for:

The phase separation-dependent FUS interactome reveals nuclear and cytoplasmic function of liquid-liquid phase separation

Stefan Reber[1,2*], Daniel Jutzi[1], Helen Lindsay[3], Anny Devoy[1], Jonas Mechtersheimer[1], Brunno Rocha Levone[4] Michal Domanski[5], Eva Bentmann[6], Dorothee Dormann[6,7,8], Oliver Mühlemann[5], Silvia M.L. Barabino[4] and Marc-David Ruepp[1,*]

[1] United Kingdom Dementia Research Institute Centre at King's College London, Institute of Psychiatry, Psychology and Neuroscience, King's College London, Maurice Wohl Clinical Neuroscience Institute, London, UK
[2] Present address: Skyhawk Therapeutics Europe GmbH, Basel, Hochbergerstrasse 60C, CH-4057, Switzerland
[3] Department of Mathematics, École polytechnique fédérale de Lausanne (EPFL), Lausanne, Switzerland
[4] Department of Biotechnology and Biosciences, University of Milano-Bicocca, Milan, Italy
[5] Department of Chemistry and Biochemistry, University of Bern, Bern, Switzerland
[6] Biomedical Center (BMC), Cell Biology, Ludwig Maximilians University Munich, Germany
[7] Munich Cluster for Systems Neurology (SyNergy), Munich, Germany
[8] present address: Johannes Gutenberg University and Institute of Molecular Biology, Mainz, Germany

Supplemental Material and methods

Plasmids

pcDNA6F-FUS, pcDNA6F-FUS P525L, pcDNA3-FLAG-eGFP, pSUPuro-scr, pSUPuro-FUS and the SCN4A reporter minigene are described elsewhere (1,2). pcDNA3.1(+)-Flag-FUS-R495X and pcDNA3.1(+)-Flag-FUS R495X-PLD27YS, have been previously described in (3) and were a kind gift of Steven McKnight. To generate pcDNA6F-FUS PLD27YS, a gene synthesis (GeneArt, Life Technologies) coding for the N-terminal domain of FUS with 27 tyrosines substituted with serines according to(3) flanked by XhoI and EcoRI restriction sites was used to clone the FUS N-term (PLD27YS) into the XhoI, EcoRI restriction sites of pcDNA6F-FUS (thereby replacing the wild type FUS N-term). To clone pcDNA6F-FUS PLD27YS P525L the aforementioned strategy was used and the FUS N-term (PLD27YS) was cloned into the XhoI, EcoRI restriction sites of pcDNA6F-FUS P525L. To generate pcDNA6F-FUS PLD27YS SV40NLS, the sequence coding for FUS PLD27YS was PCR amplified from pcDNA6F-FUS PLD27YS using the CloneAmp HiFi PCR Premix (639298, Clontech) with the forward primer 5'- ATAGGGAGACCCAAGCTGGCTAG-3' and the reverse primer introducing the SV40NLS (SV40 nuclear localisation signal) 5'-GATTGGGCCCTTCACTTGTCCTCCACTTTGCGTTTCTTTTTGGGATACGGCCTCTCCCTGCGATCC-3'. The PCR product was digested with XhoI and ApaI and cloned into the XhoI, ApaI site of pcDNA6f-FUS (replacing the coding sequence for FUS). To generate pcDNA6F-GFP-GSG15-FUS and pcDNA6F-eGFP-GSG15-FUS P525L, the sequence coding for the N-terminal FLAG tag was removed from pcDNA6F-FUS and pcDNA6F-FUS P525L, respectively and was replaced with the coding sequence for eGFP followed by a GSG15 linker which was ordered as a gene synthesis (General Biosystems) and

cloned into the XbaI, XhoI sites. The pEGFP-c1-E1A plasmid was generated according to (4). In brief the E1A minigene fragment was synthesized as DNA string (GeneArt, Life Technologies) and cloned into the BglII and SalI sites of pEGFP-C1. GB1-TwinStrep-RFP was ordered as gene synthesis (General Biosystems), cloned into the BamHI and NotI sites of pcDNA3.1(+).

qPCR Primers

Primer	5'-sequence-3'
qPCR endogenous FUS fwd	AGCGGTGTTGGAACTTCG
qPCR endogenous FUS rev	GACTGCTCTGCTGGGAATAG
qPCR β-actin fwd	TCCATCATGAAGTGTGACGT
qPCR β-actin rev	TACTCCTGCTTGCTGATCCAC
qPCR SCN4A total fwd	CAAGGGCAAGGCCATCTTC
qPCR SCN4A total rev	GCATGGATGAGCACCTTGATG
qPCR SCN4A spliced fwd	ACAAGGGCAAGGCCATCTTC
qPCR SCN4A unspliced rev	CATGCTGAACAGCGCATGG
qPCR E1A total fwd	GGATCACTCTCGGCATGGAC
qPCR E1A total rev	AGACTGGCGGCCATTTCTTC
qPCR E1A 9S isoform fwd	GCATGGACGAGCTGTACAAG
qPCR E1A 9S isoform rev	GTTCAGACACAGGACCTCTTC
qPCR E1A 12S isoform fwd	CTTGGGTCCGGTTTCTATGC
qPCR E1A 12S isoform rev	TCAGACACAGGACCCTCTTC
qPCR E1A 13S isoform fwd	CATTATCACCGGAGGAATACG
qPCR E1A 13S isoform rev	TCAGACACAGGACTGTAGAC

Antibodies and recombinant proteins

The polyclonal rabbit anti-FUS antibody, the Y12 monoclonal antibody, the rabbit anti-CPSF6 (also CFIm68) and rabbit anti-NUDT21 (also CFIm25) are described elsewhere (5-7). Additional antibodies used for this study: mouse anti-FUS (4H11) (sc-47711, Santa Cruz), mouse anti-FLAG M2 antibody (F1804, Sigma-Aldrich), rabbit anti-FLAG (14793S, Cell Signaling Technology), mouse anti-tyrosine tubulin (T9028, Sigma-Aldrich), mouse anti-VDAC1 (ab14734, abcam), mouse anti-TOM20 (sc-17764, Santa Cruz), rabbit anti-PARP1 (ab227244, abcam), rabbit anti-Lig3 (ab125434, abcam), rabbit anti-hnRNPA2B1 (ab31645, abcam), mouse anti-cytochrome c (sc-13156, Santa Cruz), mouse anti-GAPDH (sc-32233, Santa Cruz), mouse anti-hSNF2H (also SMARCA5) (sc-365727, Santa Cruz), mouse anti-Brg-1 (also SMARCA4) (sc-17796, Santa Cruz), rabbit anti-hnRNPH (A300-511A, Bethyl Laboratories), mouse anti-hnRNPA1 (sc-56700, Santa Cruz), mouse anti-SNRPC (5C9) (also U1C) (sc-101548, Santa Cruz), mouse anti-SNRPA SNRPA (BJ-7) (also U1A) (sc-101149, Santa Cruz), rabbit anti-GFP (ab6556, abcam), goat anti-GFP (AB0020-200, SICGEN), rabbit anti-histone H3 (4499, Cell Signaling Technology), mouse anti-RNAPII (CTD4H8) (05-623B, Milipore), mouse anti-G3BP (611126, BD Transduction Laboratories), goat anti-TIAR (sc-1749, Santa Cruz), goat anti-TIA-1 (C-20, Santa Cruz), rabbit anti-GFP (A-11122, Invitrogen), donkey anti-goat IRDye800CW (926-32214, LI-COR Biosciences), goat anti-mouse IRDye800CW (925-32210, LI-COR), goat anti-rabbit IRDye800CW (926-32211, LI-COR), goat anti-mouse IRDye680LT (926-68020, LI-COR), goat anti-rabbit IRDye680LT (926-68021, LI-COR), donkey anti-mouse IRDye680LT (926-68022, LI-COR Biosciences), donkey anti-rabbit AF488 (R37118, Thermo Fisher), donkey anti-goat AF568 (A-11057, Thermo Fisher), goat anti-rabbit AF488 (A27034, Thermo Fisher), goat anti-mouse AF546 (A-11003, Thermo Fisher), donkey anti-

rabbit AF-546 (A10040, Thermo Fisher), donkey anti-mouse AF-488 (A21202, Thermo Fisher). Recombinant TwinStrep-GFP was purchased from IBA Lifesciences (2-1007-005).

Fluorescence Recovery After Photobleaching (FRAP)

Two days prior to the photobleaching, HeLa FUS-KO cells were transiently transfected with either pcDNA6F-GFP-GSG15-FUS or pcDNA6F-GFP-GSG15-FUS P525L using Lipofectamine 2000 diluted in OptiMEM. Prior to the FRAP experiment, 5 x 10^4 cells were plated onto a 35-mm glass-bottom dish and a phenol red-free medium was used. A Nikon Eclipse Ti A1 confocal microscope attached to a chamber for controlled temperature (37°C), humidity and CO_2 (5%) was used. Images were taken using the 100x objective, 5x optical zoom and a 512 x 512-pixel resolution. To capture images, a speed of 2 frames per second (pixel dwell time 0.7 μs) was used. The pinhole was set to 2 μm to obtain strong fluorescence and low laser transmission (3%) was used to avoid photobleaching. FRAP experiments were done either in a nuclear/cytoplasmic spontaneously generated granules or in a side of the nucleus/cytoplasmic compartment using a validated protocol (8). Three control images were taken before photobleaching, which was done by irradiating the selected area 10 times at nominal 100% laser transmission using the 488 laser. After bleaching, a series of images were captured every 1 second for a total of 30 seconds. The fluorescence intensity from an untransfected background was subtracted from each raw fluorescence value. Data was then normalised to account for the irreversible loss of fluorescent molecules caused by the bleaching. For each time point, the relative fluorescence intensity of bleached area (Fa) is divided by the relative intensity of a transfected but unbleached area (Fb). To rescale the data in terms of percentage of initial fluorescence, the previous value is multiplied by the initial relative fluorescence intensity of a transfected but unbleached area (Fib) divided by the initial relative fluorescence intensity of the bleached area (Fia). The formula used was: $F = (Fa/Fb)*(Fib/Fia)$.

DSP/DTME Crosslinked Immunoprecipitation.

Four 75% confluent 15 cm^2 dishes with 293T cells were transfected with 12 ug pcDNA6F-FUS P525L using TransIT-LT1 (Mirus Bio) according to manufacturer's instructions. 24 hours post transfection medium was exchanged. 48 hours post transfection, 2 plates were washed twice with PBS supplemented with 0.1M $CaCl_2$ and 1 mM $MgCl_2$. Cells were then crosslinked for 30 minutes at room temperature by incubation with 25 ml of PBS supplemented with 0.1 M $CaCl_2$, 1 mM $MgCl_2$, 0.5 mM DSP (dithiobis(succinimidyl propionate), Thermo Fisher Scientific), and 0.5 mM DTME (dithiobismaleimidoethane, Thermo Fisher Scientific). To quench unreacted crosslinker, the crosslinking solution was aspirated, and cells were incubated for 15 minutes at room temperature with PBS supplemented with 0.1 M $CaCl_2$ and 1 mM $MgCl_2$ and 0.2 M glycine. The remaining uncrosslinked 2 plates were washed once with ice-cold PBS, harvested using Trypsin/EDTA, and counted using the Countess II automated cell counter (Thermo Fisher Scientific). Total lysates were generated by incubating cells with RIPA buffer supplemented with Halt protease Inhibitor (0.5 x f.c), 2 mM $MgCl_2$, 100 ug/ml RNAse A (Sigma Aldrich), 20 U Supernuclease/ml f.c (Sino Biological), and 6.25 U Cyanase/ml f.c. (Serva Electrophoresis GmbH) - 1 ml per 1x10^7 cells - followed by 30 minutes incubation on ice with occasional vortexing and passing the crosslinked lysate through a 23G and 27¾ G needle. 1.6% of the

Lysates were mixed with 2 x LDS buffer (total protein). The remaining lysate was cleared by centrifugation (15 minutes 12'000 x g) to generate total extracts. 1.6% of extracts were mixed with 2 x LDS buffer (input) and the insoluble fraction (pellet) was resuspended with 1x LDS buffer with the same volume as the initial lysate. 200 µl Flag M2 beads (Sigma Aldrich), washed 2x in 1x TBS supplemented with 0.05% Igepal-CA630, were incubated with 12 ml total extract and incubated on a rotating wheel for 1.5 hours at 4 °C. Beads were washed 5 times 5 minutes with TBS-0.1 % Igepal CA630 (Sigma Aldrich), followed by elution of the immunoprecipitated material from the FLAG-M2 beads by incubation for 30 minutes at 4 °C using 2 mg/ml FLAG peptide f.c. in 200 µl TBS-0.1 % Igepal CA630. Eluates (IP) were mixed 1:1 with 2 x LDS buffer. Samples were denatured for 10 minutes at 70°C and loaded on a 4-12 % NuPage gel, usgin either 1x MOPS or 1x MES running buffer, followed by western blotting.

Duolink Proximity Ligation Assay

60 – 80 % confluent HeLa cells on 6-well plates were transfected with 1 µg of either WT FUS-FLAG or P525L FUS-FLAG expression construct using Dogtor according to manufacturer's instructions. 24 h after transfection the cells were split on uncoated 12-well microscope slides (ibidi, 81201). 48 h after transfection the cells were fixed for 10 min at 37°C with 4% PFA in PBS supplemented with 4% sucrose, 5 mM $MgCl_2$ and 10 mM EGTA before being permeabilized with 0.3% Triton X-100 in PBS for 10 min at RT. The PLA was then performed on the cells using the Duolink in Situ Red kit (DUO92008, Sigma Aldrich) according to manufacturer's protocol using mouse MINUS (DUO82004, Sigma Aldrich)) and rabbit PLUS (DUO82002, Sigma Aldrich) probes. Immunofluorescence was performed alongside the PLA using identical fixation, permeabilization, primary antibody incubation and wash steps, while the secondary antibody incubation step was conducted for 100 min at 37°C. All primary antibodies were used in a 1:100 dilution. The cells were then mounted using Duolink in Situ mounting medium with DAPI (DUO82040, Sigma Aldrich) and subsequently imaged using a non-confocal Ti-E epifluorescence microscope, Nikon.

Cytoplasmic vs mitochondrial (membrane) and soluble vs insoluble fractionations

60-80 % confluent HEK293T cells in T25 flasks were harvested using Trypsin/EDTA and washed once with ice cold PBS. Cytoplasmic/mitochondrial fractionation was performed according to (9) with slight modifications: Cells were re-suspended in 400 µl buffer A (150 mM NaCl, HEPES pH7.5, 25 µg/ml Digitonin (Sigma Aldrich, D141), 1 M Hexylene glycol (Sigma Aldrich, 112100), 1X Halt™ Protease Inhibitor Cocktail) and incubated for 10 min at 4 °C head over tail on a rotor. Samples were centrifuged for 10 min at 2,000 g at 4 °C and the supernatant (cytoplasmic fraction) was transferred to a new 2 ml Eppendorf tube. The pellet was subsequently washed 2 x with buffer A (5 min 2,000 g centrifugation steps between washes discarding the supernatant). Thereafter, the pellet was re-suspended in buffer B (150 mM NaCl, HEPES pH7.5, 1 % (v/v) NP-40, 1 M Hexylene glycol, 1X Halt™ Protease Inhibitor Cocktail) and incubated for 30 min on ice. Samples were centrifuged for 10 min at 7,000 g at 4 °C and the supernatant (mitochondrial fraction) was transferred to a new 2 ml Eppendorf tube. To concentrate the samples, proteins were precipitated with 4x volumes of acetone for 1 h at – 20 °C and centrifugation

for 1 h at 16,000 g at 4 °C. Protein pellets were re-suspended in 50 µl 1X LDS-loading buffer and boiled for 5 min at 95 °C.

Supplemental Figures

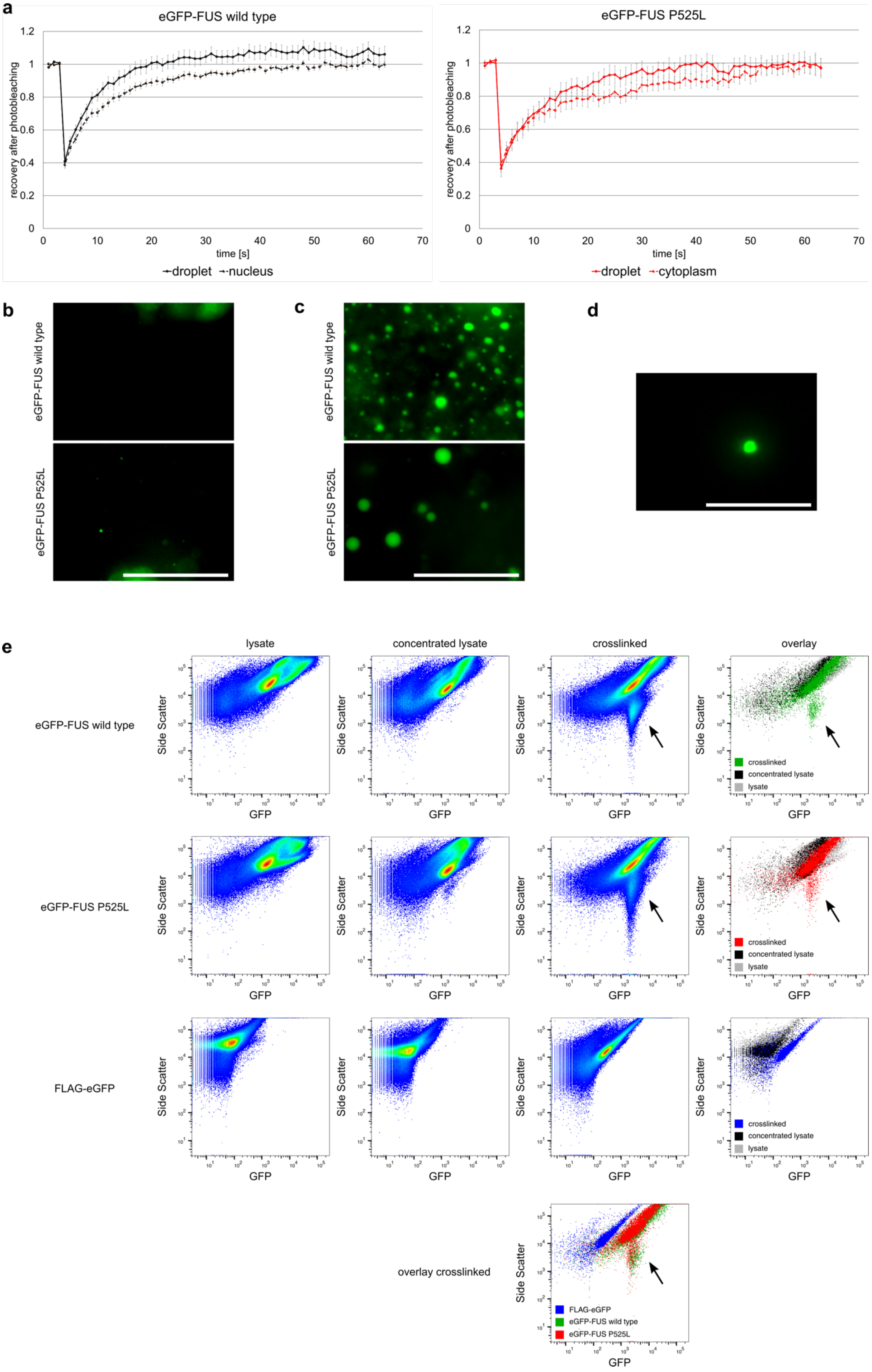
a
eGFP-FUS wild type
eGFP-FUS P525L
recovery after photobleaching
time [s]
droplet
nucleus
droplet
cytoplasm
b
c
d
eGFP-FUS wild type
eGFP-FUS P525L
e
lysate
concentrated lysate
crosslinked
overlay
eGFP-FUS wild type
eGFP-FUS P525L
FLAG-eGFP
Side Scatter
GFP
crosslinked
concentrated lysate
lysate
overlay crosslinked
FLAG-eGFP
eGFP-FUS wild type
eGFP-FUS P525L

Supplementary Figure 1

Analysis of eGFP-FUS droplet formation by FRAP, fluorescence microscopy and flow cytometry. **a** FUS WT and FUS P525L spontaneously generated granules display liquid-like properties. Photobleaching of a WT granule versus a photobleached nuclear area nearby (left) and photobleaching of a P525L granule versus a photobleached cytoplasmic area nearby (right). Two biological replicates were performed with ten individual cells being bleached in each of them (n = 20). **b** Picture of cell lysates before concentrating showing the absence of FUS droplets. Scale bar = 25 µm. **c** Concentrated cell lysates before crosslinking showing the formation of eGFP-FUS droplets. eGFP-FUS droplets of varying size and cell debris are visible. Scale bar = 25 µm. **d** Picture of purified droplet after sorting. Scale bar = 25 µm. **e** Flow cytometric analysis of lysates (first column), concentrated lysates (second column) and crosslinked lysates (third column) from cells which were expressing eGFP-FUS (top row), eGFP-FUS P525L (second row) or FLAG-eGFP (third row). The last column shows the overlay of each row. The fourth row shows the overlay of the third column. The population of droplets, which are only stable after crosslinking, are indicated with black arrows.

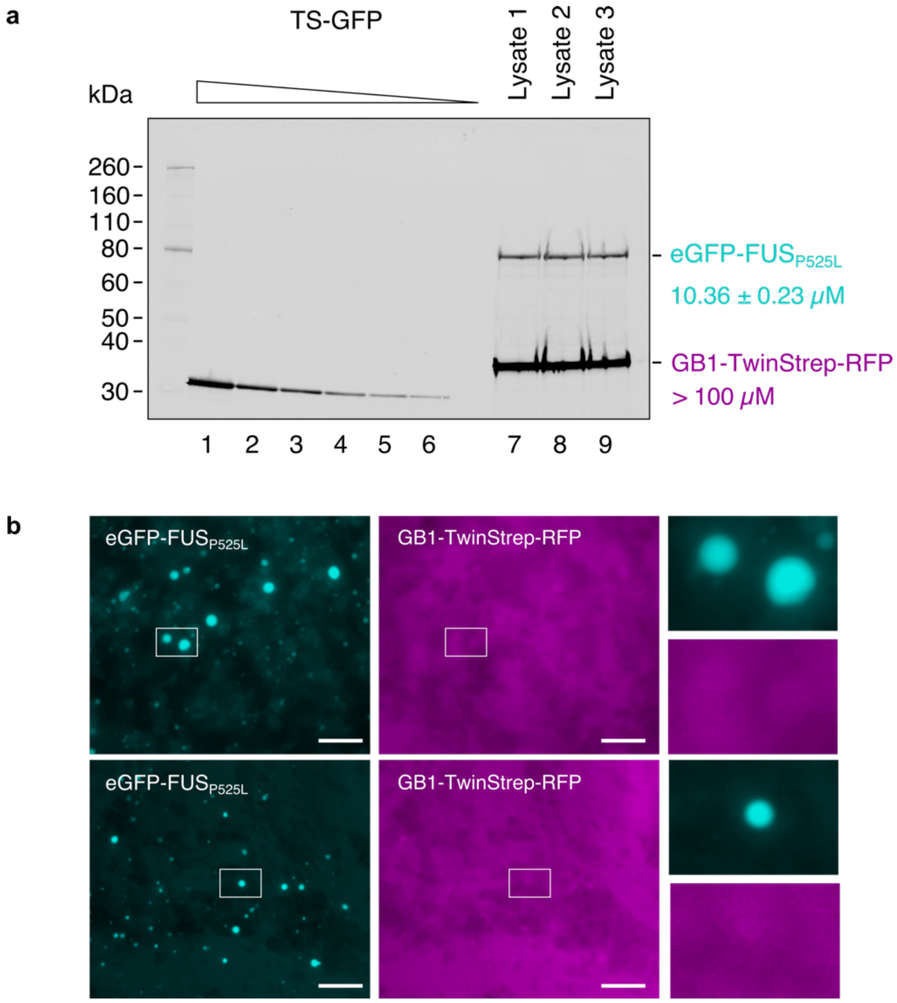

Supplementary Figure 2

eGFP-FUS droplet formation using GB1-TwinStrep-RFP as soluble internal marker. **a** Western blot of recombinant TwinStrep-tagged GFP (lanes 1-6, corresponding to 1.0, 0.5, 0.25, 0.12, 0.06 and 0.03 µg of protein) and three lysates from HEK293T cells transfected with eGFP-FUS P525L and GB1-TwinStrep-RFP (lanes 7-9). The anti-GFP antibody recognises both eGFP and RFP. eGFP-FUS concentrations were determined by densitometry and are displayed as mean ± standard deviation, n = 3. **b** Fluorescence microscopy pictures of eGFP-FUS P525L (cyan) and GB1-TwinStrep-RFP (magenta) in 2-fold concentrated cell lysates. Scale bar = 20 µm.

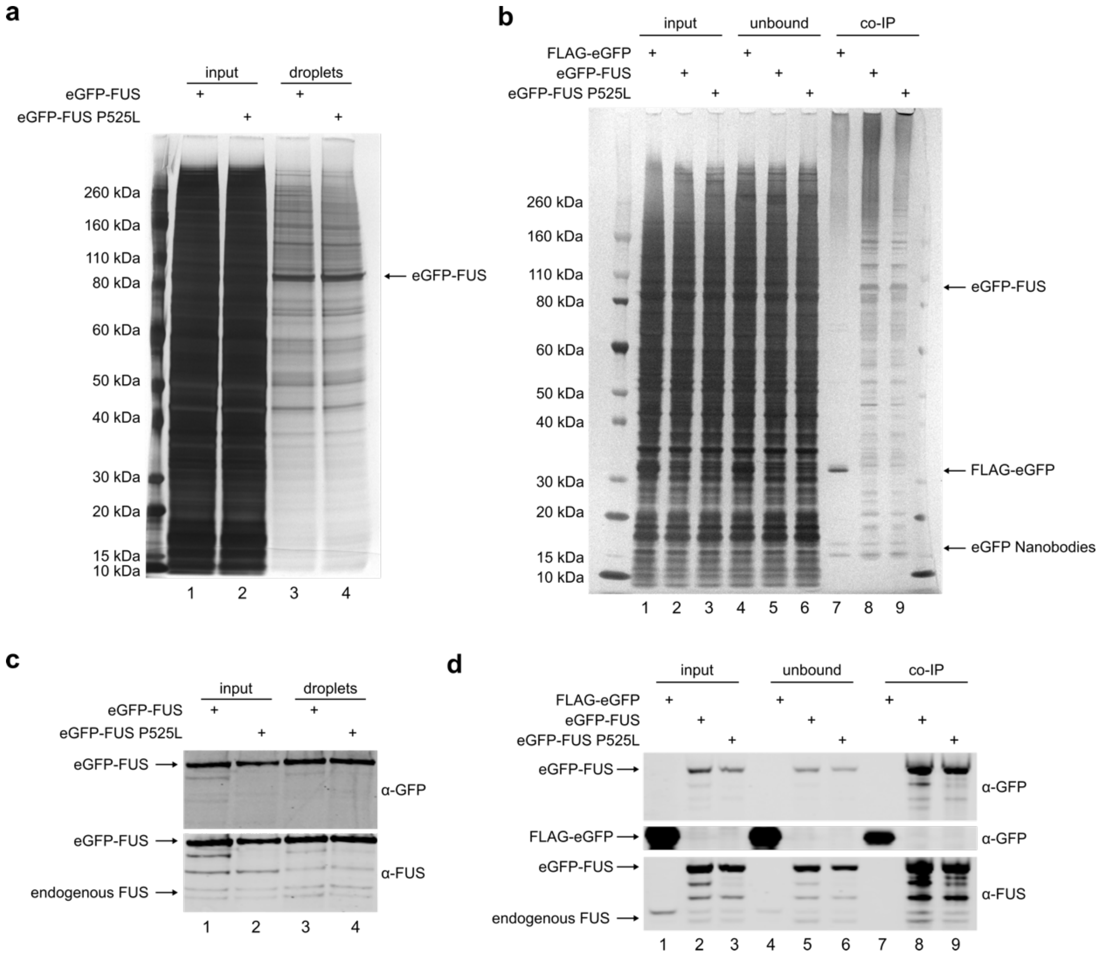

Supplementary Figure 3

Silver stainings and western blots of droplet purification and co-immunoprecipitation experiments separated on 4-12 % Bis-Tris gels. **a** Silver staining of input (lane 1-2) and purified droplets (lane 3-4). Arrow indicates eGFP-FUS and eGFP-FUS P525L, respectively. **b** Silver staining of input (lane 1-3), unbound fractions (4-6) and co-immunoprecipitated proteins together with FLAG-eGFP (lane 7), eGFP-FUS (lane 8) and eGFP-FUS P525L (lane 9). Arrows indicate baits and eGFP Nanobodies which were partially boiled off the beads (2 bands at > 15 kDa). **c** Western blot of samples shown in a. Proteins were transferred on a nitrocellulose membrane and the membrane was subsequently probed with anti-GFP (top panel) and anti-FUS (bottom panel) antibodies. **d** Western blot of samples shown in b. The nitrocellulose membrane was treated as in c.

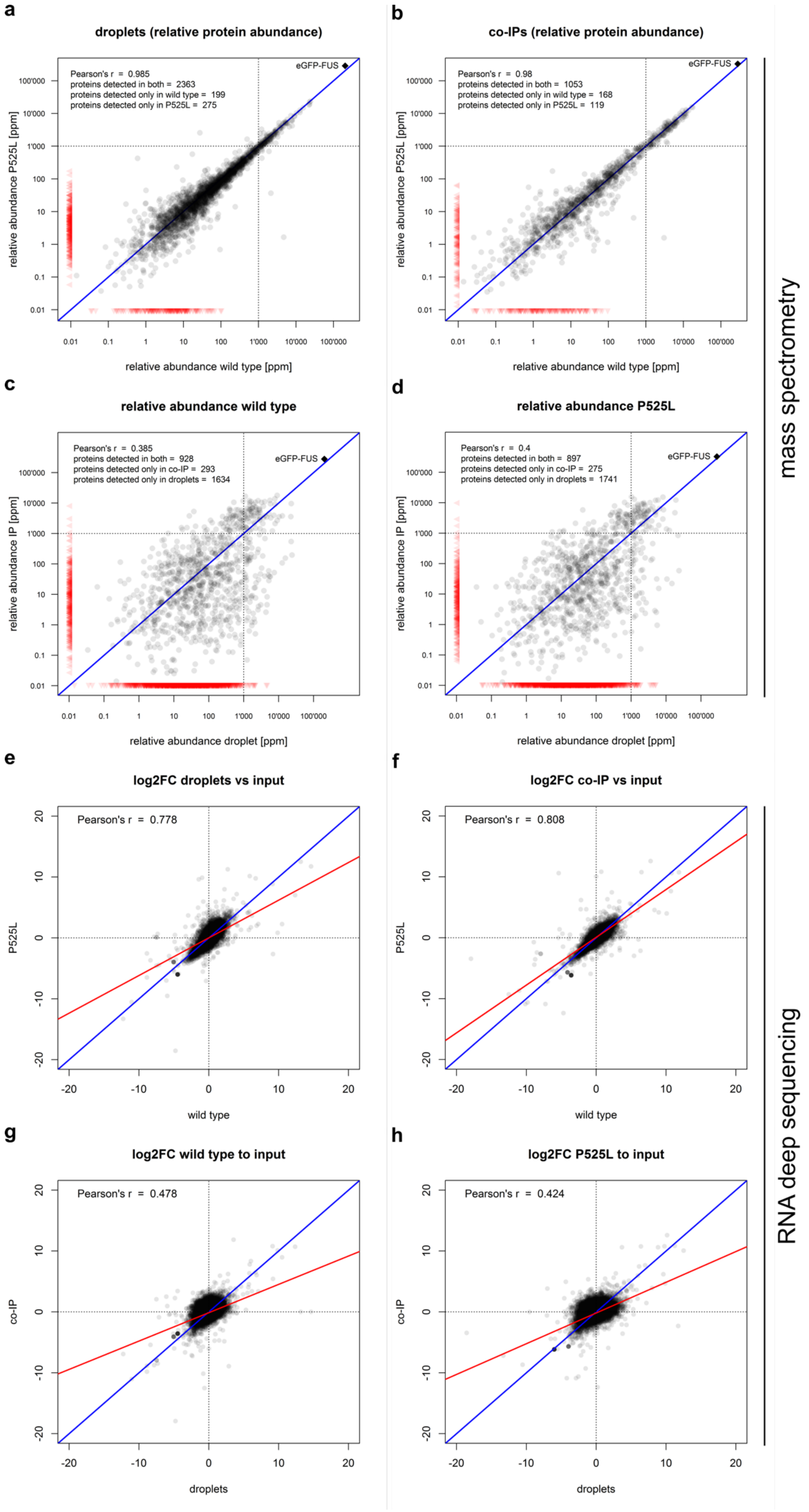
a
droplets (relative protein abundance)
Pearson's r = 0.985
proteins detected in both = 2363
proteins detected only in wild type = 199
proteins detected only in P525L = 275
eGFP-FUS
relative abundance P525L [ppm]
relative abundance wild type [ppm]
b
co-IPs (relative protein abundance)
Pearson's r = 0.98
proteins detected in both = 1053
proteins detected only in wild type = 168
proteins detected only in P525L = 119
eGFP-FUS
relative abundance P525L [ppm]
relative abundance wild type [ppm]
c
relative abundance wild type
Pearson's r = 0.385
proteins detected in both = 928
proteins detected only in co-IP = 293
proteins detected only in droplets = 1634
eGFP-FUS
relative abundance IP [ppm]
relative abundance droplet [ppm]
d
relative abundance P525L
Pearson's r = 0.4
proteins detected in both = 897
proteins detected only in co-IP = 275
proteins detected only in droplets = 1741
eGFP-FUS
relative abundance IP [ppm]
relative abundance droplet [ppm]
e
log2FC droplets vs input
Pearson's r = 0.778
P525L
wild type
f
log2FC co-IP vs input
Pearson's r = 0.808
P525L
wild type
g
log2FC wild type to input
Pearson's r = 0.478
co-IP
droplets
h
log2FC P525L to input
Pearson's r = 0.424
co-IP
droplets
mass spectrometry
RNA deep sequencing

Supplementary Figure 4

Plots showing correlations between proteins (a-d) and RNAs (e-h) detected in quantitative mass spectrometry and RNAdeep sequencing, respectively. Note that there is a high correlation between wild type FUS and P525L FUS samples within the same experiment (a-b and e-f) while a much lower or no correlation can be observed if droplet and co-IP samples are compared (c-d and g-h). **a** Plot showing relative abundance in parts per million (ppm) of all proteins which were detected in wild type (x-axis) and P525L FUS (y-axis) droplets. The dotted lines label 1,000 ppm (= 0.1 %). The blue line depicts perfect correlation. Black dots represent proteins that were detected in both samples. Red triangles represent proteins that were detected in one sample only. The bait (eGFP-FUS) is represented as a black square. Pearson's correlation coefficient was calculated on the non-logarithmic values and the bait (eGFP-FUS) was excluded from the calculation. **b** Same plot as in a, but showing relative abundances of proteins detected in the co-IP experiments. **c** Same plot as in a, but comparing droplet and co-IP experiment of wild type FUS samples. **d** Same as in a, but comparing droplet and co-IP experiment of P525L FUS samples. **e** Plot showing the log2 fold change of RNA isolated from droplets compared to the input together with wild type (x-axis) and P525L FUS (y-axis). The blue line depicts perfect correlation, the red line the actual correlation between the samples. Each black dot represents one gene (n = 13,096) **f** Same as in e, but showing log2 fold changes of RNAs isolated in the co-IP experiment. **g** Same plot as in e, but comparing droplet and co-IP experiments of wild type FUS samples. **h** Same as in a, but comparing droplet and co-IP experiment of P525L FUS samples.

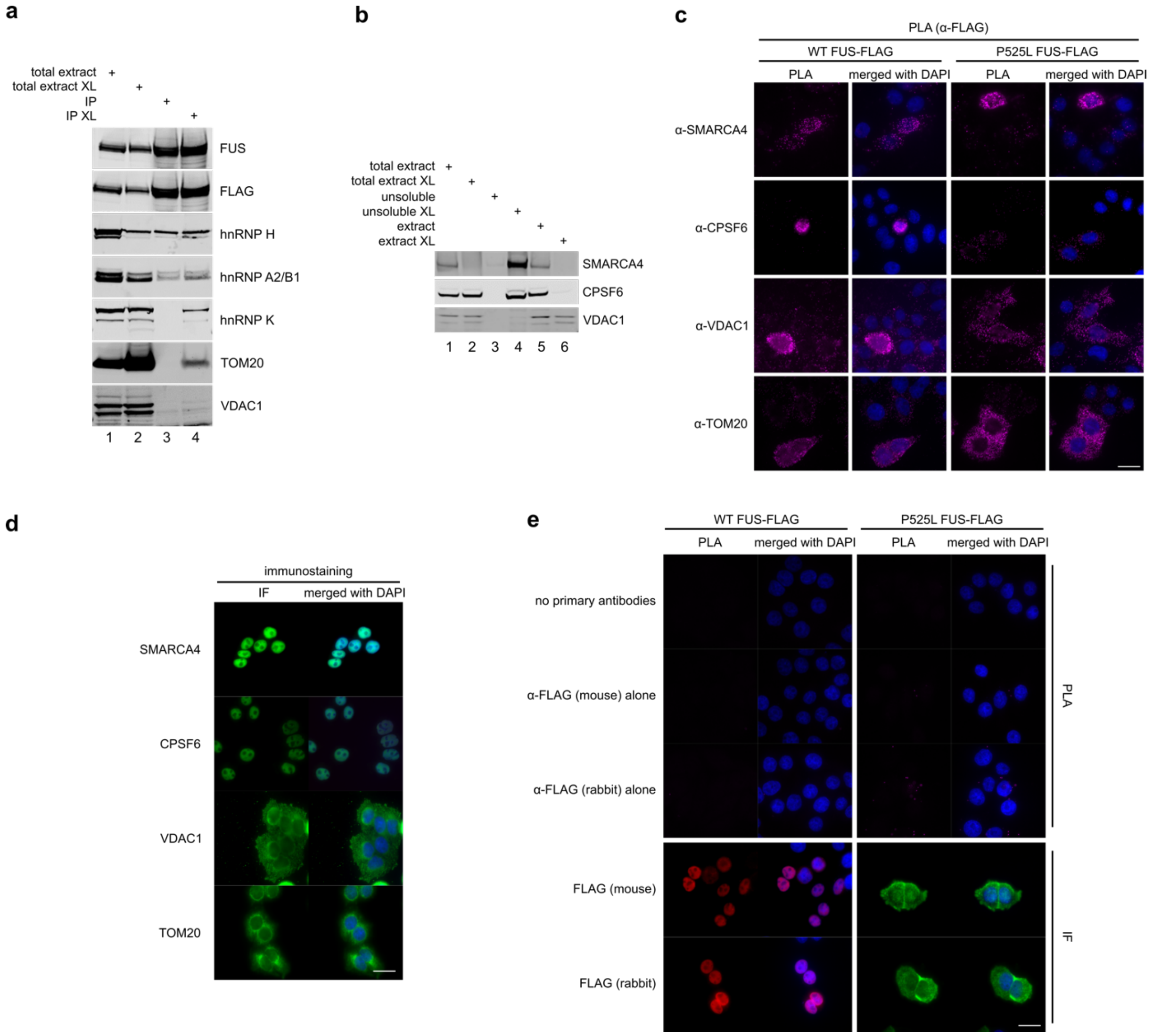

Supplementary Figure 5

Validation of interactors described in Figure 2. **a** Western blot analysis of input (total extract) and co-immunoprecipitated proteins from 293T cells transfected with FLAG-FUS P525L with or without DSP/DTME in-cell crosslinking (XL) prior to extract preparation **b** Western blot analysis of total lysate, unsoluble pellet and soluble total extract from FLAG-FUS P525L transfected 293T cells with or without DSP/DTME in-cell crosslinking (XL) prior to lysate and extract preparation. **c** Proximity ligation assay on HeLa cells either transfected with wild type (WT) or P525L FLAG-tagged FUS. Shown are the PLA signals only (lane 1, lane 3) and the overlays of the PLA signal with DAPI counterstaining (lane 2, lane 4) for FLAG with either SMARCA4 (first row), CPSF6 (second row), VDAC1 (third row), and TOM20 (fourth row). Scale bar = 25 µm. **d** Antibody validation on HeLa cells. Cells were counterstained using DAPI. Scale bar = 25 µm. **e** PLA specificity control with HeLa cells either transfected with WT or P525L FLAG-tagged FUS and subjected to the PLA protocol incubated without primary antibody (first row) or solely with anti-FLAG antibody produced in either mouse (second row) or rabbit (third row). Shown are the PLA signals only (lane 1, lane 3) and the overlays of the PLA signal with DAPI counterstaining (lane 2, lane 4). Lower part of the subfigure shows antibody validation of the anti-FLAG antibodies. Cells were counterstained using DAPI. Scale bar = 25 µm.

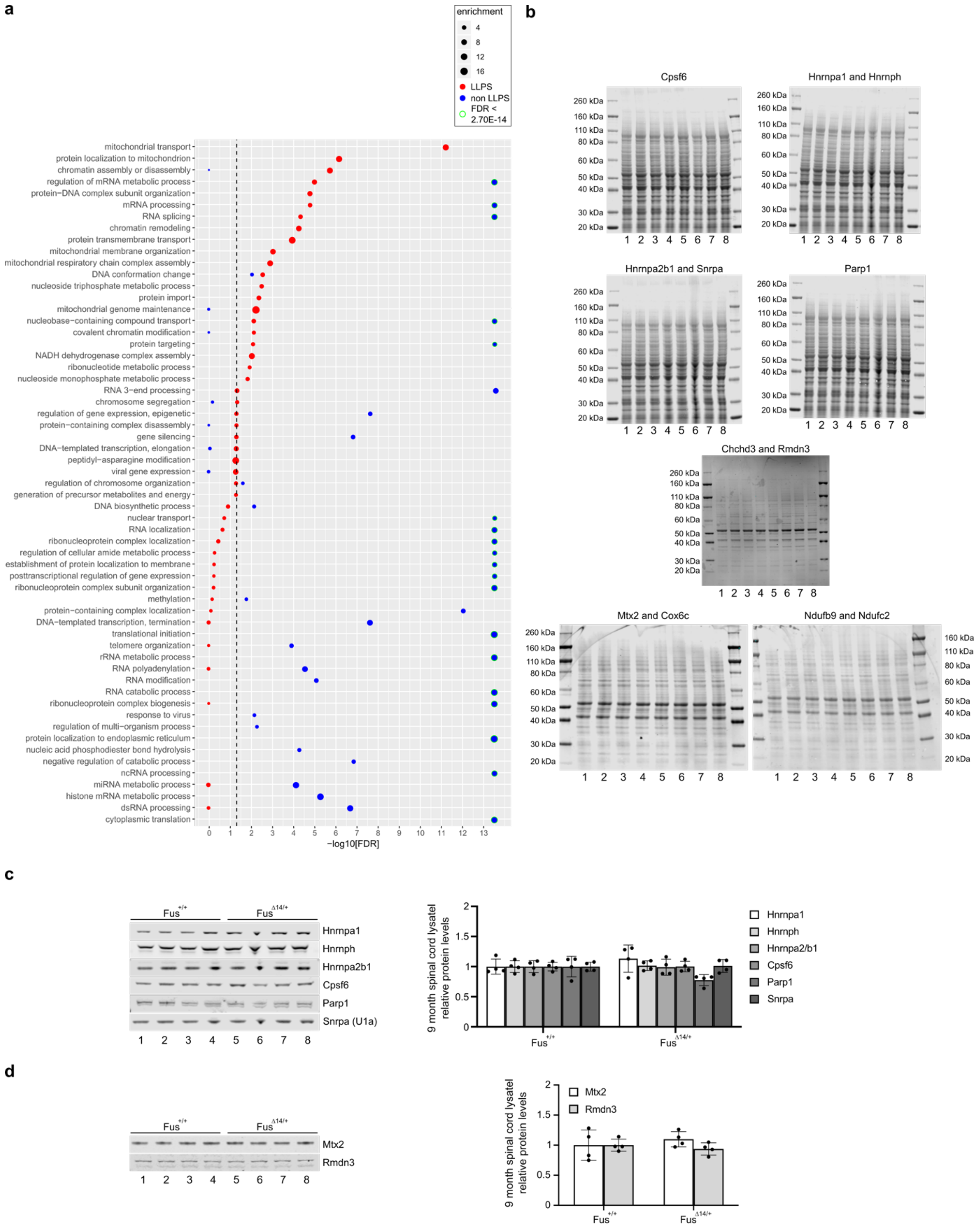

Supplementary Figure 6

A GO term analysis of LLPS-specific FUS interactors (red) and proteins interacting with FUS under non-LLPS conditions (blue). The dotted line indicates a FDR < 0.05. All significantly enriched GO terms for both FUS interactomes are shown. Missing dots indicate that the respective GO term was not detected in the respective FUS interactome. **b** Total protein stainings used for quantification of protein levels and normalization in Figure 2g and Supplementary Figure 4c and d. Note that while membranes were routinely blocked after total protein staining (and scan) were performed, the total protein staining for Chchd3 and Rmdn3 was performed after the membrane was blocked. **c** Western blot (left) and quantification (right) of proteins involved in RNA splicing and chromatin remodelling. **d** Western blot (left) and quantification (right) of proteins with mitochondrial function.

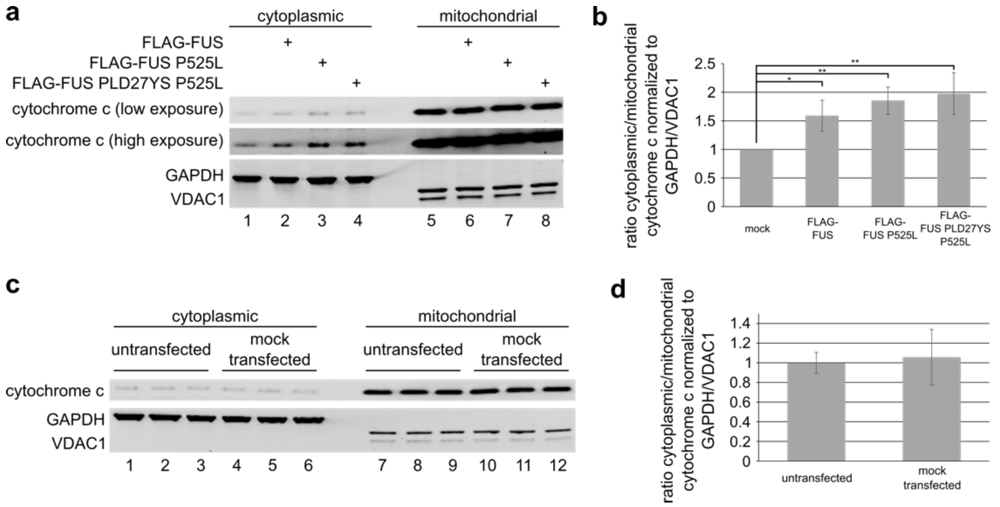

Supplementary Figure 7

LLPS is not required for cytoplasmic FUS toxicity. **a** Cytoplasmic/mitochondrial fractionation of untransfected, FLAG-FUS, FLAG-FUS P525L and FLAG-FUS PLD27YS P525L transfected HEK293T cells, respectively. Cytoplasmic (lanes 1-4) and mitochondrial (lanes 5-8) fractions were analysed by western blotting using anti cytochrome c antibody (top and middle row). Cytochrome c signal is shown in two different exposures. GAPDH and VDAC1 (lower row) served as controls for cytoplasmic and mitochondrial fractions, respectively. **b** Quantification of cytochrome c levels in a. Shown are the ratios of cytoplasmic to mitochondrial cytochrome c relative to the control. Average values and standard deviations from five biological replicates are shown. Single and double asterisk indicate a p-values of < 0.05 and < 0.01, respectively. **c** Cytoplasmic/mitochondrial fractionation of untransfected and mock transfected HEK293T cells, respectively. Cytoplasmic (lanes 1-6) and mitochondrial (lanes 7-12) fractions were analysed by western blotting using anti cytochrome c antibody (top row). GAPDH and VDAC1 (lower row) served as controls for cytoplasmic and mitochondrial fractions, respectively. **d** Quantification of cytochrome c levels in a. Shown are the ratios of cytoplasmic to mitochondrial cytochrome c relative to the first replicate of the untransfected condition. Average values and standard deviations from three biological replicates are shown.

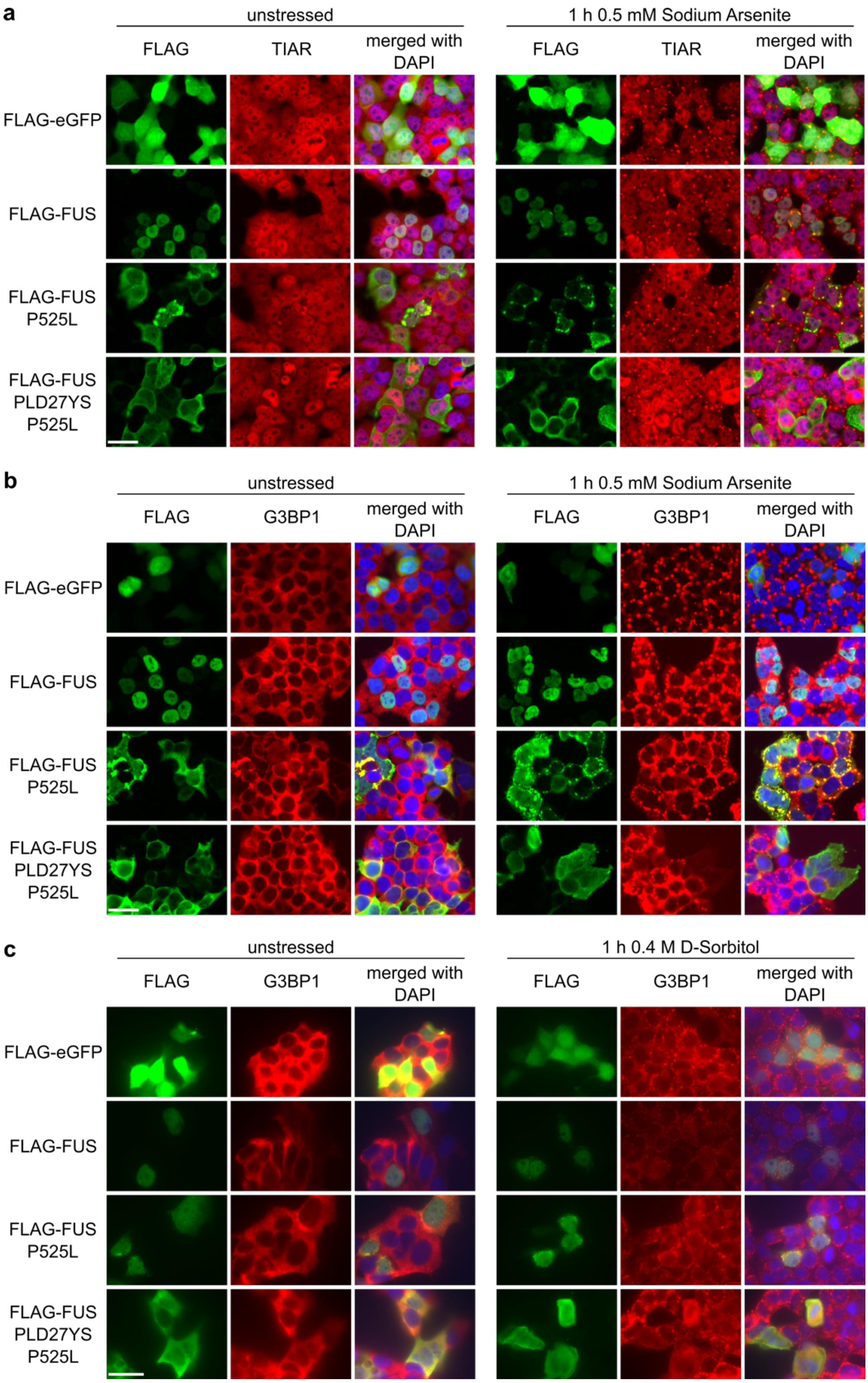

Supplementary Figure 8

a Immunostaining of unstressed (left) and stressed (right) HEK293T cells transiently transfected with the indicated FLAG-FUS constructs. In addition, a FLAG-eGFP control was included. Cells were stained for FLAG (green) and the stress granule marker TIAR (red). Cells were counterstained using DAPI. Scale bar = 30 µm. **b** Same as in a, cells were stained for the stress granule marker G3BP1 (red). Scale bar = 30 µm **c** Same as in b, but with osmotic instead of oxidative stress using D-sorbitol. Scale bar = 30 µm.

	Gene	fold change droplets vs co-IP	FDR	abundance in droplets [ppm]	abundance in co-IP [ppm]
mediator complex	MED17	Inf	0.2484996	0.390832	0
	MED14	Inf	0.2289878	0.294638	0
	MED12	Inf	0.08755816	1.94505	0
	MED24	Inf	0.03518069	0.8961252	0
	MED23	Inf	0.2138961	3.347663	0
	MED1	Inf	0.02249834	0.7839777	0
RNA Polymerase I	POLR1A	9.746799	0.003839289	19.69436	2.020597
	POLR1B	0.460853	0.05772821	2.961577	6.426294
	POLR1C	7.097573	0.000389579	115.1318	16.22129
	POLR1E	Inf	0.05967345	6.569027	0
RNA Polymerase II	POLR2A	18.07852	0.006004162	28.18542	1.559056
	POLR2B	6.908363	0.03257622	30.4071	4.401492
	POLR2C	Inf	0.003309718	24.0212	0
	POLR2E	Inf	0.01547834	54.65555	0
	POLR2H	Inf	0.03534031	16.97889	0
	POLR2I	0	0.03399944	0	21.13068
RNA Polymerase III	POLR3A	240.4713	0.02766891	12.2363	0.05088465
	POLR3B	43.79838	0.08332625	6.157186	0.1405802
	POLR3C	Inf	0.009059487	5.164592	0
	POLR3E	Inf	0.4954505	0.5857161	0

Supplementary Figure 9 – data extracted from Supplementary Table S1
Mass spectrometry data extracted from Supplementary Table S1. Protein components of the mediator complex and RNA Polymerase II are more abundant under LLPS (droplets) than under non-LLPS (co-IP) conditions together with FUS. Note that most of the proteins were not detected in the non-LLPS condition (abundance of 0). Interestingly, the same appears to be true for RNA Pol I and especially RNA Pol III.

a

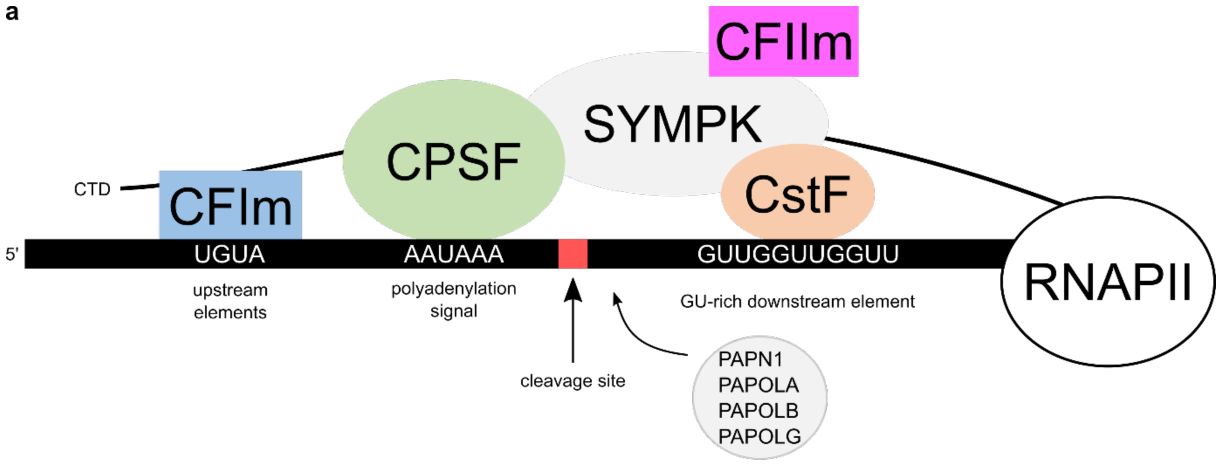

b

	Gene	fold change droplets vs co-IP	FDR	abundance in droplets [ppm]	abundance in co-IP [ppm]
**Cleavage factor Im (CFIm)**	NUDT21	7.954754	6.54E-04	1810.49	227.5985
	CPSF6	9.126611	9.64E-05	1136.175	124.4904
	CPSF7	52.14858	1.77E-03	552.5726	10.59612
**Cleavage and polyadenylation specificity factor (CPSF)**	CPSF1	0.1574387	2.91E-02	24.45202	155.3114
	CPSF2	0.2187055	2.09E-02	49.26569	225.2604
	CPSF3	0.3904558	9.57E-02	34.78517	89.08864
	CPSF4	0	9.03E-02	0	27.19969
	WDR33	0.09601287	1.10E-02	5.513332	57.42285
	FIP1L1	0.256468	4.76E-03	32.53274	126.8491
**additional factors**	SYMPK	0.9332519	1.62E-01	44.44383	47.62254
	PABPN1	0.1978951	2.35E-04	49.18344	248.5329
	PAPOLA	Inf	3.09E-02	3.763685	0
	PAPOLB	not detected			
	PAPOLG	not detected			
**Cleavage stimulation factor (CstF)**	CSTF1	1.960767	1.16E-02	38.3178	19.54225
	CSTF2	1.420094	1.74E-01	27.6281	19.45513
	CSTF2T	0.5119832	6.99E-01	0.396281	0.7740115
	CSTF3	2.538518	1.13E-02	31.86525	12.5527
**Cleavage factor IIm (CFIIm)**	PCF11	0.8762395	6.67E-01	1.675131	1.911727
	CLP1	not detected			

Supplementary Figure 10 – data extracted from Supplementary Table S1
a Scheme summarizing the protein complexes involved in 3'-end processing of pre-mRNA according to (10). **b** Mass spectrometry data extracted from Supplementary Table S1. While most components of the 3'-end processing machinery shown no clear preference for LLPS (droplets) or non-LLPS (co-IP) FUS, the three members of CFIm are strongly enriched under LLPS conditions.

Supplementary video

Time lapse video showing spontaneous fusion of eGFP-FUS droplets in a concentrated cell lysate. Pictures of concentrated cell lysates were acquired using a wide-field fluorescence Leica DMI6000 B microscope in one second intervals (time stamp in seconds indicated).

High resolution version of Figures 2a and 2b

Figure 2a

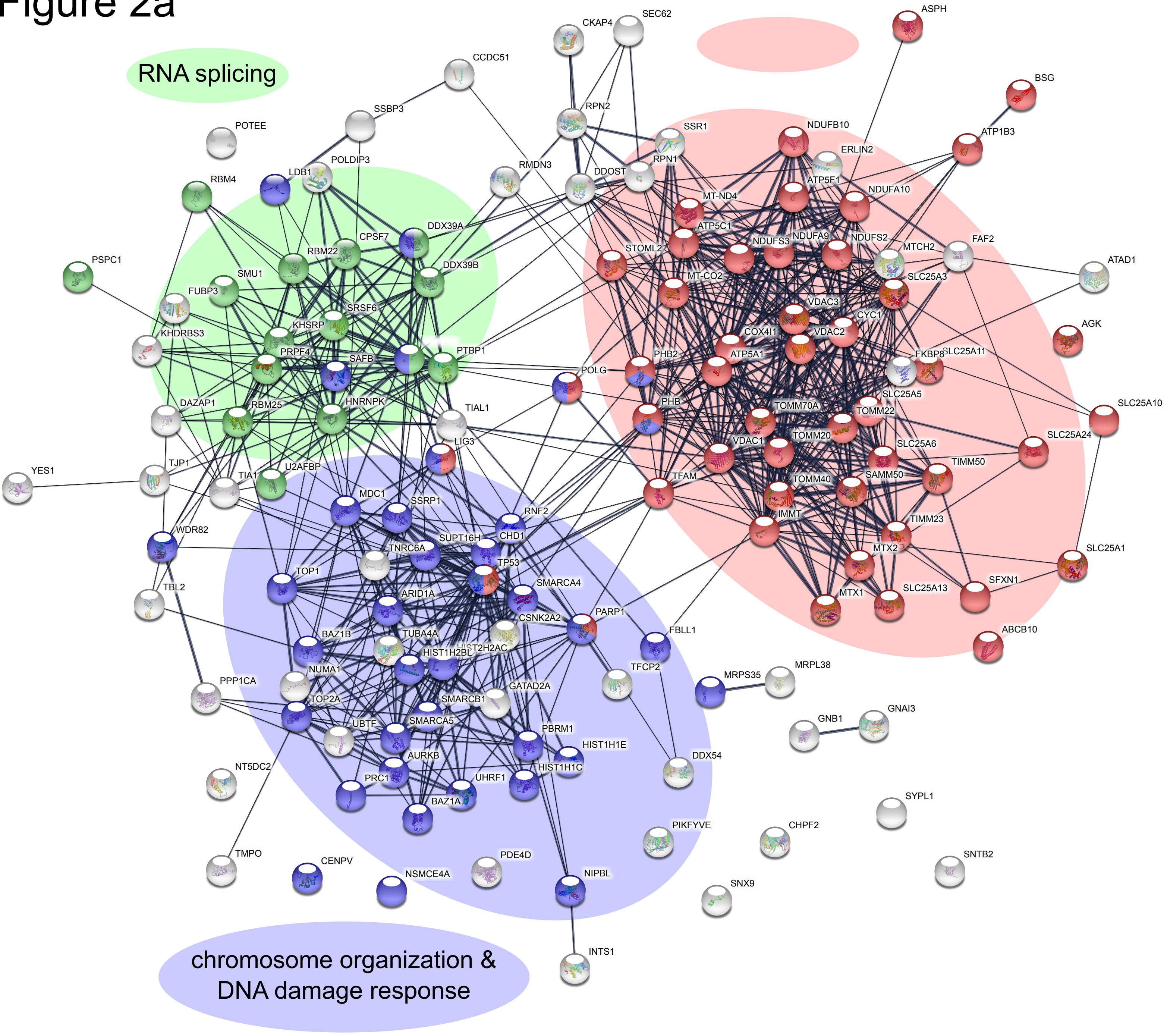

RNA splicing
chromosome organization &
DNA damage response
ASPH
CKAP4
SEC62
BSG
CCDC51
SSBP3
RPN2
POTEE
SSR1
NDUFB10
ATP1B3
ERLIN2
POLDIP3
RMDN3
RPN1
LDB1
RBM4
DDOST
ATP5F1
NDUFA10
MT-ND4
DDX39A
CPSF7
ATP5C1
NDUFS3
NDUFA9
NDUFS2
MTCH2
FAF2
RBM22
STOML2
PSPC1
DDX39B
ATAD1
SMU1
MT-CO2
SLC25A3
FUBP3
SRSF6
VDAC3
CYC1
KHSRP
AGK
KHDRBS3
COX4I1
VDAC2
PRPF4
PTBP1
PHB2
ATP5A1
FKBP8
SLC25A11
SAFB
POLG
SLC25A5
DAZAP1
HNRNPK
PHB
TOMM70A
SLC25A10
RBM25
TIAL1
TOMM22
TOMM20
SLC25A24
LIG3
VDAC1
SLC25A6
TJP1
U2AFBP
YES1
TIA1
TFAM
SAMM50
TIMM50
MDC1
TOMM40
SSRP1
RNF2
IMMT
TIMM23
WDR82
SUPT16H
CHD1
TNRC6A
MTX2
SLC25A1
TP53
TOP1
SMARCA4
SFXN1
TBL2
ARID1A
MTX1
SLC25A13
PARP1
CSNK2A2
BAZ1B
TUBA4A
FBLL1
ABCB10
HIST1H2BL
TFCP2
MRPL38
NUMA1
MRPS35
PPP1CA
GATAD2A
TOP2A
SMARCB1
GNAI3
UBTF
SMARCA5
GNB1
PBRM1
HIST1H1E
AURKB
DDX54
NT5DC2
HIST1H1C
PRC1
UHRF1
SYPL1
BAZ1A
PIKFYVE
CHPF2
SNTB2
TMPO
CENPV
PDE4D
NSMCE4A
NIPBL
SNX9
INTS1

Figure 2b

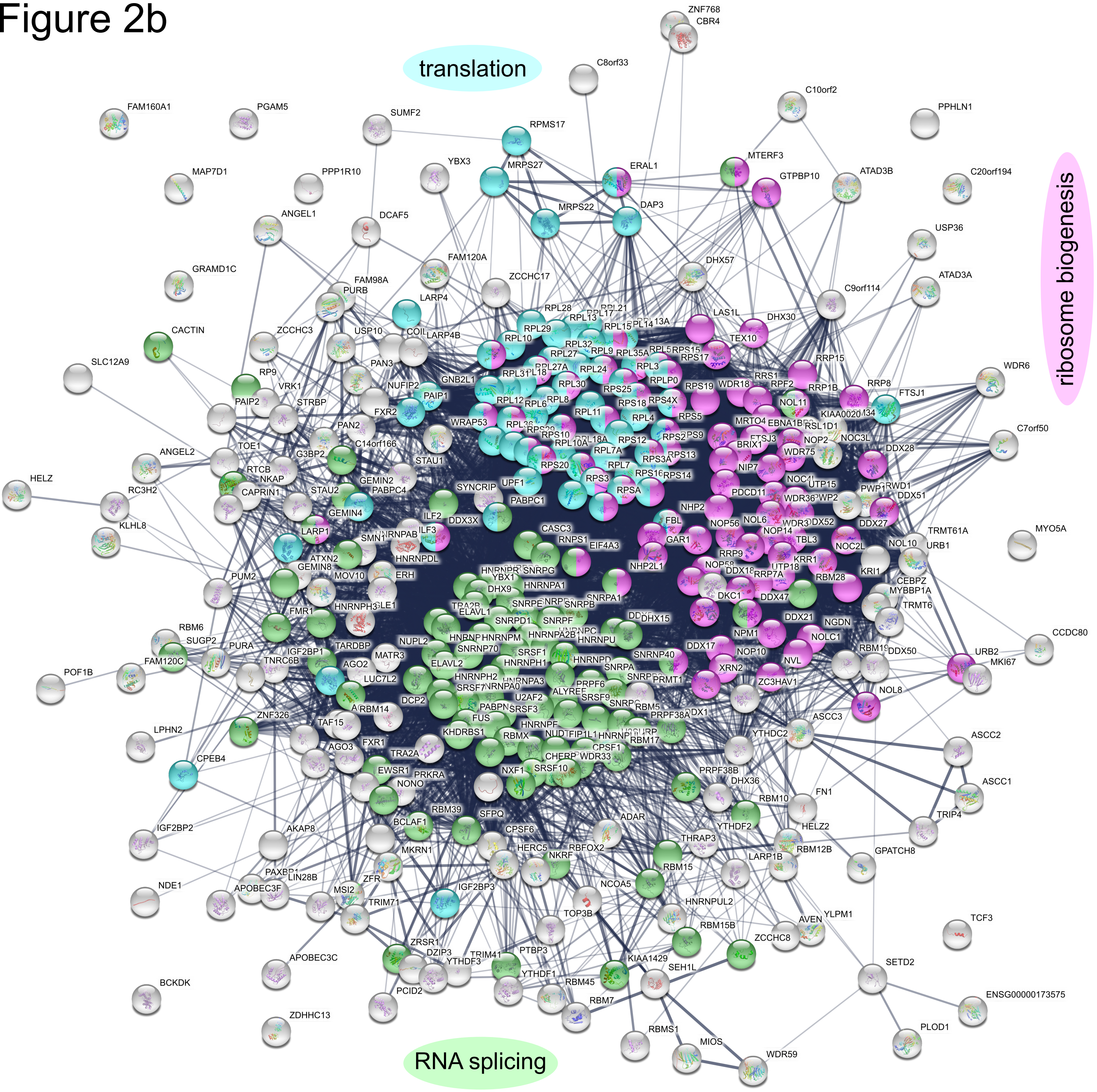

translation
ribosome biogenesis
RNA splicing
ZNF768
CBR4
C8orf33
C10orf2
PPHLN1
FAM160A1
PGAM5
SUMF2
RPMS17
MAP7D1
PPP1R10
YBX3
MRPS27
ERAL1
MTERF3
GTPBP10
ATAD3B
C20orf194
ANGEL1
DCAF5
MRPS22
DAP3
USP36
ATAD3A
GRAMD1C
FAM120A
ZCCHC17
DHX57
C9orf114
FAM98A
PURB
LARP4
RPL28
RPL21
RPL13
RPL17
LAS1L
DHX30
CACTIN
ZCCHC3
USP10
COIL
LARP4B
RPL29
RPL10
RPL15
RPL14
RPL13A
TEX10
WDR6
SLC12A9
RPL32
RPL27
RPL9
RPL35A
RPS15
RPS17
RRP15
RP9
PAN3
RPL27A
RPL24
RPL3
RRS1
RPF2
RRP1B
RRP8
FTSJ1
VRK1
NUFIP2
GNB2L1
RPL31
RPL18
RPL30
RPS25
RPLP0
RPS19
WDR18
PAIP2
STRBP
PAIP1
RPL12
RPL6
RPL8
RPS18
RPS4X
NOL11
FXR2
RPL11
RPL4
RPS5
KIAA0020
C7orf50
PAN2
WRAP53
RPL36
MRTO4
EBNA1BP2
RSL1D1
TOE1
C14orf166
RPS10
RPS12
RPS2
RPS9
FTSJ3
NOP2
NOC3L
ANGEL2
G3BP2
STAU1
RPL10A
RPL7A
BRIX1
WDR75
DDX28
RTCB
RPS20
RPL7
RPS3A
RPS13
NIP7
HELZ
NKAP
GEMIN2
RPS16
RPS14
RC3H2
CAPRIN1
STAU2
PABPC4
SYNCRIP
UPF1
RPS3
RPSA
UTP15
PDCD11
WDR36
DDX51
PABPC1
NHP2
GEMIN4
ILF2
DDX3X
FBL
NOP56
NOL6
DDX52
DDX27
KLHL8
LARP1
ILF3
CASC3
NOP14
TBL3
TRMT61A
MYO5A
RNPS1
EIF4A3
GAR1
NOC2L
NOL10
URB1
ATXN2
HNRNPDL
RRP9
GEMIN8
PUM2
MOV10
ERH
SNRPG
NHP2L1
UTP18
KRR1
KRI1
YBX1
HNRNPA1
DDX18
RRP7A
RBM28
CEBPZ
DHX9
MYBBP1A
SNRPA1
DKC1
DDX47
TRMT6
FMR1
ELAVL1
SNRPB
RBM6
SNRPD1
SNRPF
DHX15
DDX21
NGDN
CCDC80
SUGP2
PURA
NUPL2
HNRNPM
HNRNPU
NPM1
NOLC1
IGF2BP1
TARDBP
MATR3
SNRNP70
SRSF1
DDX17
NOP10
DDX50
URB2
POF1B
FAM120C
TNRC6B
AGO2
ELAVL2
HNRNPH1
HNRNPD
SNRNP40
XRN2
NVL
MKI67
SNRPA
LUC7L2
HNRNPH2
PRMT1
ZC3HAV1
SRSF7
PRPF6
NOL8
DCP2
U2AF2
ALYREF
SRSF9
ZNF326
RBM14
PABPN1
SRSF3
RBM5
PRPF38A
LPHN2
TAF15
FUS
ASCC3
CPEB4
AGO3
FXR1
KHDRBS1
RBMX
FIP1L1
RBM17
YTHDC2
ASCC2
TRA2A
CPSF1
WDR33
EWSR1
PRKRA
NXF1
SRSF10
PRPF38B
DHX36
ASCC1
NONO
RBM10
FN1
RBM39
SFPQ
ADAR
TRIP4
IGF2BP2
AKAP8
BCLAF1
YTHDF2
HELZ2
CPSF6
THRAP3
MKRN1
HERC5
RBFOX2
RBM12B
GPATCH8
NKRF
LARP1B
RBM15
NDE1
LIN28B
APOBEC3F
ZFR
MSI2
IGF2BP3
NCOA5
TRIM71
HNRNPUL2
TOP3B
TCF3
AVEN
YLPM1
RBM15B
ZCCHC8
ZRSR1
DZIP3
TRIM41
PTBP3
KIAA1429
APOBEC3C
YTHDF3
SEH1L
SETD2
YTHDF1
BCKDK
RBM45
RBM7
ENSG00000173575
PCID2
ZDHHC13
RBMS1
PLOD1
MIOS
WDR59

Supplemental References

1. Reber, S., Stettler, J., Filosa, G., Colombo, M., Jutzi, D., Lenzken, S.C., Schweingruber, C., Bruggmann, R., Bachi, A., Barabino, S.M. *et al.* (2016) Minor intron splicing is regulated by FUS and affected by ALS-associated FUS mutants. *EMBO J*, **35**, 1504-1521.
2. Rufener, S.C. and Muhlemann, O. (2013) eIF4E-bound mRNPs are substrates for nonsense-mediated mRNA decay in mammalian cells. *Nat Struct Mol Biol*, **20**, 710-717.
3. Kato, M., Han, Tina W., Xie, S., Shi, K., Du, X., Wu, Leeju C., Mirzaei, H., Goldsmith, Elizabeth J., Longgood, J., Pei, J. *et al.* (2012) Cell-free Formation of RNA Granules: Low Complexity Sequence Domains Form Dynamic Fibers within Hydrogels. *Cell*, **149**, 753-767.
4. Kino, Y., Washizu, C., Aquilanti, E., Okuno, M., Kurosawa, M., Yamada, M., Doi, H. and Nukina, N. (2011) Intracellular localization and splicing regulation of FUS/TLS are variably affected by amyotrophic lateral sclerosis-linked mutations. *Nucleic acids research*, **39**, 2781-2798.
5. Raczynska, K.D., Ruepp, M.D., Brzek, A., Reber, S., Romeo, V., Rindlisbacher, B., Heller, M., Szweykowska-Kulinska, Z., Jarmolowski, A. and Schumperli, D. (2015) FUS/TLS contributes to replication-dependent histone gene expression by interaction with U7 snRNPs and histone-specific transcription factors. *Nucleic acids research*, **43**, 9711-9728.
6. Lerner, E.A., Lerner, M.R., Janeway, C.A., Jr. and Steitz, J.A. (1981) Monoclonal antibodies to nucleic acid-containing cellular constituents: probes for molecular biology and autoimmune disease. *Proceedings of the National Academy of Sciences of the United States of America*, **78**, 2737-2741.
7. Ruegsegger, U., Blank, D. and Keller, W. (1998) Human pre-mRNA cleavage factor Im is related to spliceosomal SR proteins and can be reconstituted in vitro from recombinant subunits. *Mol Cell*, **1**, 243-253.
8. Day, C.A., Kraft, L.J., Kang, M. and Kenworthy, A.K. (2012) Analysis of protein and lipid dynamics using confocal fluorescence recovery after photobleaching (FRAP). *Curr Protoc Cytom*, **Chapter 2**, Unit2 19.
9. Baghirova, S., Hughes, B.G., Hendzel, M.J. and Schulz, R. (2015) Sequential fractionation and isolation of subcellular proteins from tissue or cultured cells. *MethodsX*, **2**, 440-445.
10. Gruber, A.R., Martin, G., Keller, W. and Zavolan, M. (2014) Means to an end: mechanisms of alternative polyadenylation of messenger RNA precursors. *Wiley interdisciplinary reviews. RNA*, **5**, 183-196.
